# The development of metabolic endotoxemia is dependent on the type of sweetener and the presence of saturated fat in the diet

**DOI:** 10.1080/19490976.2020.1801301

**Published:** 2020-08-17

**Authors:** Mónica Sánchez-Tapia, Aaron W. Miller, Omar Granados-Portillo, Armando R. Tovar, Nimbe Torres

**Affiliations:** a Departamento de Fisiología de la Nutrición, Instituto Nacional de Ciencias Médicas y Nutrición Salvador Zubirán, Ciudad de México, México; b Department of Inflammation and Immunity, Lerner Research Institute, Cleveland Clinic, Cleveland, OH, USA

**Keywords:** Metabolic endotoxemia, natural and artificial sweeteners, high-fat diet, gut microbiota, metagenomics, TLR4, occludin, short-chain fatty acids, energy expenditure, GPR43, GPR41

## Abstract

Fat and sweeteners contribute to obesity. However, it is unknown whether specific bacteria are selectively modified by different caloric and noncaloric sweeteners with or without a high-fat diet (HFD). Here, we combined extensive host phenotyping and shotgun metagenomics of the gut microbiota to investigate this question. We found that the type of sweetener and its combination with an HFD selectively modified the gut microbiota. Sucralose and steviol glycosides led to the lowest α-diversity of the gut microbiota. Sucralose increased the abundance of *B. fragilis* in particular, resulting in a decrease in the abundance of occludin and an increase in proinflammatory cytokines, glucose intolerance, fatty acid oxidation and ketone bodies. Sucrose+HFD showed the highest metabolic endotoxemia, weight gain, body fat, total short chain fatty acids (SCFAs), serum TNFα concentration and glucose intolerance. Consumption of sucralose or sucrose resulted in enrichment of the bacterial genes involved in the synthesis of LPS and SCFAs. Notably, brown sugar and honey were associated with the absence of metabolic endotoxemia, increases in bacterial gene diversity and anti-inflammatory markers such as IL-10 and sIgA, the maintenance of glucose tolerance and energy expenditure, similar to the control group, despite the consumption of an HFD. These findings indicate that the type of sweetener and an HFD selectively modify the gut microbiota, bacterial gene enrichment of metabolic pathways involved in LPS and SCFA synthesis, and metabolic endotoxemia associated with different metabolic profiles.

## Introduction

The trillions of microorganisms that inhabit the human gut, referred to as the gut microbiota, perform extensive metabolic activities essential to maintaining host homeostasis and health. Variations in the composition of the gut microbiota induce metabolic changes that may result in alterations in host phenotype.^1^ Diet is a major environmental factor involved in shaping the composition and function of the gut microbiota and therefore its impact on host health and disease. Although the external environment plays an important role in shaping the microbial community, the host can affect the microbial ecosystem through its immune system, which also exerts an impact on the fecal metabolic content. While the study of the 16S rRNA gene does not fully capture the metabolic activity of the gut microbiome, whole metagenomic shotgun sequencing broadly captures the genetic milieu of the gut microbiota and thus allows broader profiling of the metabolic potential present. Observational studies comparing fecal microbiota from healthy subjects to patients with different types of metabolic diseases strongly suggest that the gut microbiota plays a significant role in the etiology and development of obesity and diabetes that is accompanied by a low-grade inflammation known as metabolic endotoxemia.^2,3^

Currently, the increase in obesity rates worldwide has been associated with an increase in the intake of energy-dense foods that are not only high in fat but also high in sugar.^4^ To combat this trend without compromising our preference for sweet foods, noncaloric sweeteners (NCS) have been developed to reduce caloric intake, and consumption of other natural sweeteners such as brown sugar or honey has been promoted. However, studies in both mice and humans have described the effects of some NCS, mainly saccharin, sucralose and aspartame on host glucose intolerance.^5^ This effect is accompanied by altered intestinal communities associated with obesity and diabetes and microbial gene composition, indicating that NCS impact microbial function and modify the production of several metabolites, including short-chain fatty acids (SCFAs). SCFAs are ligands of G-protein-coupled receptors (GPRs) 41 and 43 in intestinal, adipose and pancreatic tissues and several types of immune cells, indicating that they play an important role in the crosstalk between the gut and peripheral tissues as well as in protective immunity and inflammation.^6^ However, there is scarce evidence regarding whether NCS or some other caloric sweeteners could generate metabolic endotoxemia or regulate SCFA production and the immune system of the host. Early evidence has suggested that both sugar and saturated fat consumption contribute to inflammatory processes and the pathogenesis of obesity-induced insulin resistance (Xu et al., 2003) that may be associated with the excessive production of pro-inflammatory cytokines, low-grade inflammation and glucose intolerance.

However, the specific mechanism by which caloric and noncaloric sweeteners produce glucose intolerance is not well understood. In addition, there is limited evidence regarding whether complex or less refined sweeteners, particularly honey or brown sugar, have similar effects. Thus, the aim of the present work was to study whether the type of sweetener and the presence of a high-fat diet differentially regulate host metabolism, metabolic endotoxemia, the gut microbiota and their metabolic consequences.

## Results

### The type of sweetener determines body weight gain, body fat and lean body mass

**Figure 1.**
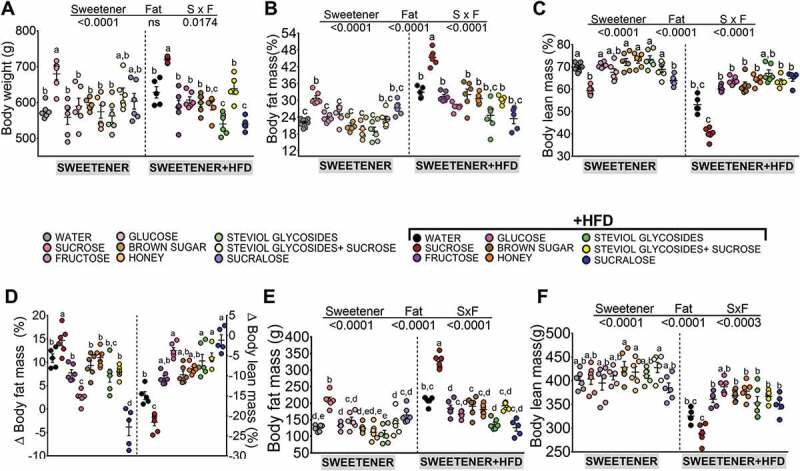
Effect of different types of sweeteners with and without a high-fat diet on body weight and body composition. (a-b) Final body weight, (c-d) % body fat mass, (e-f) % lean body mass, (g) body fat weight (g) and (h) lean body mass weight (g) in rats fed different types of sweeteners in the absence or presence of a high-fat diet. The raw data and means±SEMs are shown in each plot with n = 6–7 in each group. W = water, S = sucrose, F = fructose, G = glucose, SG = steviol glycosides, BS = brown sugar, H = honey, SV = steviol glycosides+sucrose and SU = sucralose.

To determine the effects of different types of sweeteners, we added sucrose (S), glucose (G), fructose (F), honey (H), brown sugar (BS), sucralose (SU), steviol glycosides (SG), or SG+sucrose (SV) to the drinking water of lean 6-week-old Wistar rats fed a control or high-fat diet (HFD). Interestingly, each sweetener showed different antioxidant activities, with honey, brown sugar and steviol glycosides being the sweeteners with the highest antioxidant activity (Figure S1). We used rats given drinking water with no additives as the control group. The control diet used in this study adhered to the recommendations of the American Institute of Nutrition.^7^ The groups fed different sweeteners and an HFD followed the diet described in Table S1. We first analyzed the effects of the type of sweetener when fed the control diet. Notably, at week 17, rats fed 10% sucrose gained 24.5% more weight than rats fed glucose or fructose or the rats given water with no additives. Since the group fed sucrose gained the most weight among all the groups, we compared this group with the groups fed the noncaloric sweeteners (NCS) sucralose and steviol glycosides. The sucralose group gained 11.3% less body weight than the sucrose group (Figure 1a). We also used an artificial sweetener containing steviol glycosides plus sucrose (SV). The weight gain of the steviol glycoside group was similar to that of control group (C). However, the SV group showed greater weight gain than the steviol glycosides group alone, indicating that the addition of sucrose to the steviol glycosides negated the beneficial effect of steviol glycosides on body weight (Figure 1a). We further compared the weight gain among the sucrose group and the groups fed partially refined sugars, particularly brown sugar, and complex sweeteners, such as honey. Interestingly, there was no significant difference in body weight between the control, brown sugar or honey groups (Figure 1a). Subsequently, we analyzed the weight gain of groups fed the different types of sweeteners in combination with a high-fat diet. Notably, the group fed sucrose+HFD showed the highest body weight gain, whereas those fed steviol glycosides and sucralose showed the lowest body weight gain, 25% less than the sucrose+HFD group, (Figure 1b).

Results pertaining to weight gain were consistent with those for body composition. Animals fed sucrose had 209 g of fat mass, and rats fed sucralose had 165 g of fat mass, whereas animals fed glucose or fructose had 148 g fat mass and 131.8 g fat mass, respectively. In addition, the groups with lowest body fat mass were fed steviol glycosides, honey and brown sugar at 107.5 g, 113.0 g and 125 g, respectively, whereas the control group had 125.2 g of fat mass. In the groups fed an HFD, the highest % body fat mass gain was observed in the group fed sucrose+HFD with 327 g, which was 56.4% higher than the sucrose group (Figure 1c,d,g,h). The group with the lowest body fat mass was sucralose+HFD at 23.1% less than the sucralose group (Figure 1d).

In contrast, the % of body lean mass was similar in all groups with the exception of sucrose (Figure 1e). When lean body mass expressed in grams was considered, however, all groups showed similar lean body mass (Figure 1h). The addition of an HFD reduced lean body mass in all groups, mainly in the sucrose+HFD group expressed as % of lean body mass or as grams of lean mass (figure 1f,h).

### The type of sweetener and fat differentially modify the gut microbiota

**Figure 2.**
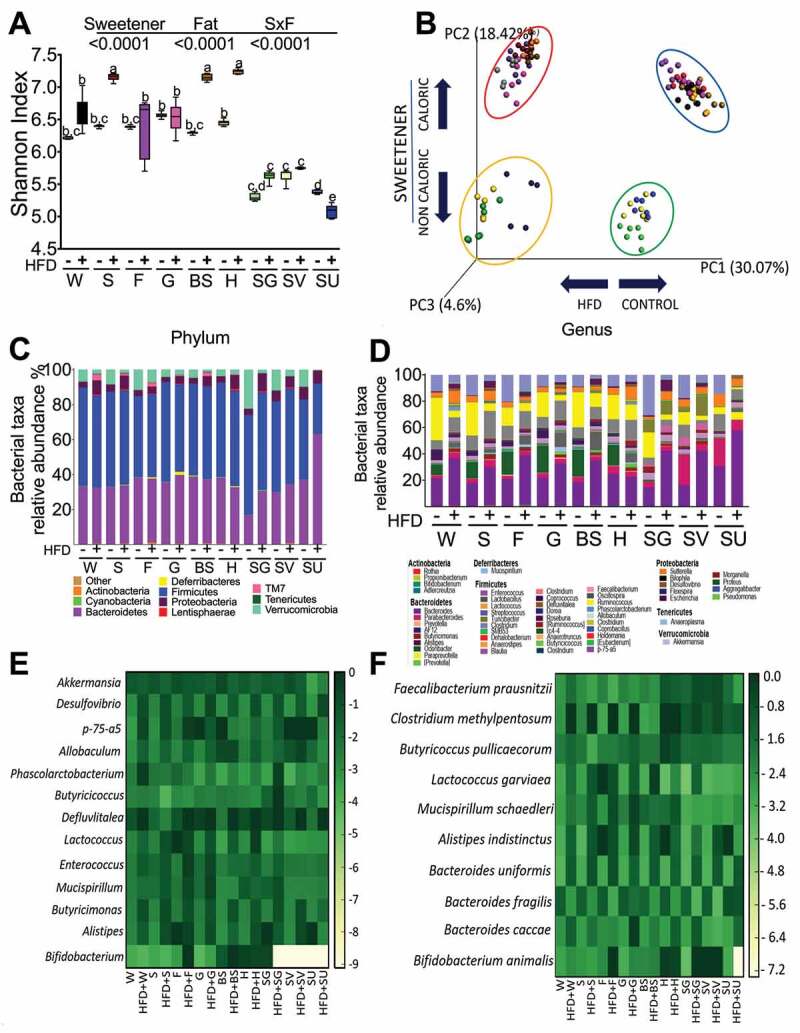
Different types of sweeteners modify the gut microbiota diversity and composition. (a) Alpha diversity by Shannon index, (b) Principal component analysis, (c) Relative abundance of the gut microbiota at the phylum and (d) genus levels, (e) Heatmap of the ten bacterial genera or (f) species with the greatest differences among groups after the consumption of different types of sweeteners with or without a high-fat diet. n = 6–7 in each group. W = water, S = sucrose, F = fructose, G = glucose, SG = steviol glycosides, BS = brown sugar, H = honey, SV = steviol glycosides+sucrose and SU = sucralose.

The increase in body weight observed in the groups fed different sweeteners could be explained in part by changes in the gut microbiota; we therefore assessed the 16S rRNA gene sequences. Overall, rats fed caloric sweeteners had more microbial diversity even in the presence of an HFD than those fed noncaloric sweeteners (Figure 2a). The PCoA analysis revealed that gut microbiota is differentially modified by the fat content in the diet and by the type of sweetener. The beta diversity analysis showed that 30.07% of the microbiota variation is explained by the presence of fat in the diet, and 18.42% of the microbiota variation is due to the type of sweetener, indicating that 48.5% of the microbiota distribution is due to the type of sweetener and the addition of fat to the diet (Figure 2b).

At the phylum level, sucrose, fructose, glucose, brown sugar, honey and sucralose showed a ratio of Bacteroidetes/Firmicutes between 0.6–0.8 with and without an HFD, whereas the steviol glycosides group had a lower ratio (0.3–0.55), and the group fed sucralose+HFD had a ratio of 2.2, indicating a significant alteration in the gut microbiota, *p* < .0001 (Figure 2c).

At the genus level, the most downregulated taxa were *Lactoccoccus, Mucispirillum*, and *Bifidobacterium* in the groups fed NCS, whereas the groups fed brown sugar and honey had increased *Bifidobacterium* with and without fat and decreased *Enterococcus* taxa. Addition of fat to the diet decreased the abundance of the *Akkermansia* genus with all sweeteners and increased the abundance of *Desulfovibrio, Enterococcus* and *Butyricimonas* (Figure 2d,e).

At the species level, the abundance of *Faecalibacterium prausnitzii* increased with honey and steviol glycosides, even with an HFD. Interestingly, *Bacteroides uniformis* and *Bacteroides caccae* increased with an HFD, regardless of the type of sweetener, while *B fragilis* showed the opposite pattern. The group fed sucralose+HFD demonstrated a significant increase in the three *Bacteroides* species. It is worth mentioning that the honey group had a high abundance of *Butyricoccus pullicaecorum*, a butyrate producer,^8^ and *Bifidobacterium animalis* associated with a reduction in adiposity^9^ (figure 2f).

Thus, we inquire how different types of sweeteners can induce different types of dysbiosis with similar metabolic abnormalities. In addressing this question, we measured changes in the functional capacity of the microbiota produced by sweeteners in combination with an HFD using a comparative shotgun metagenomics approach.

### Different sweeteners in combination with fat produce different microbial functional capacities

**Figure 3.**
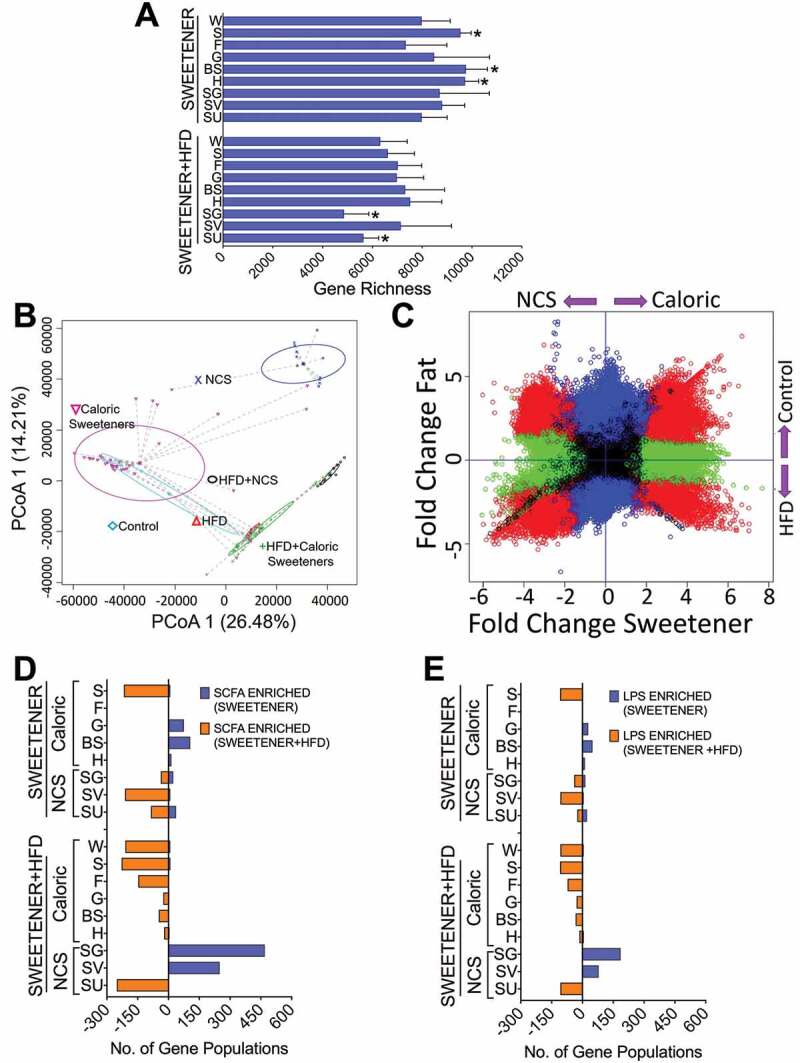
The type of sweetener and the dietary fat content modify the gene capacity of the gut microbiota. (a) Gene richness, (b) Principal component analysis, (c) Differential gene expression. All sweeteners were categorized as either NCS or caloric, and diets were categorized as HFD or Control. (d) Number of genes involved in SCFA synthesis and (e) LPS synthesis determined by metagenomic analysis after the consumption of different types of sweeteners with or without a high-fat diet. n = 6–7 in each group. W = water, S = sucrose, F = fructose, G = glucose, SG = steviol glycosides, BS = brown sugar, H = honey, SV = steviol glycosides+sucrose, SU = sucralose and NCS = noncaloric artificial sweeteners.

The shotgun analysis revealed the full metagenome of the microorganisms and the functional differences in specific pathways modified by the consumption of specific sweeteners with or without an HFD. The highest gene richness in the gut microbiota was observed in the groups fed sucrose, honey and brown sugar, whereas the lowest gene richness was observed in the groups fed sucralose+HFD and steviol glycosides+HFD (Figure 3a). The PCoA analysis revealed that the microbial community was different for both the type of sweetener and the addition of fat to the diet (Figure 3b), similar to the pattern observed in the 16S rRNA gene analysis (Figure 2b; Tables S2, S3). To identify genes that were differentially expressed between the type of sweetener and the presence of fat, gene abundance normalization and differential expression analysis were performed using the DESeq2 software package.^10^ Volcano plots were used to illustrate the overall gene expression data with an adjusted FDR *p*-value<0.05 to capture highly abundant marginal changes in gene expression (Figure 3c). The metagenomic analysis revealed that the number of genes involved in the formation of SCFAs was increased in the sucrose, sucralose and SV groups, and the presence of an HFD increased the number of these genes in all groups with the exception of the brown sugar and honey groups, even in the presence of an HFD (Figure 3d). The metagenomic analysis revealed that the groups fed a steviol glycosides+HFD, sucrose +HFD, sucralose+HFD and sucrose had the highest number of genes involved in LPS synthesis. It is important to note that the groups fed honey, brown sugar, or steviol glycosides had the lowest number of genes involved in LPS production (Figure 3e).

### Complex sweeteners maintain the intestinal epithelial length and occludin abundance and prevent metabolic endotoxemia and inflammation mediated by TLR4

**Figure 4.**
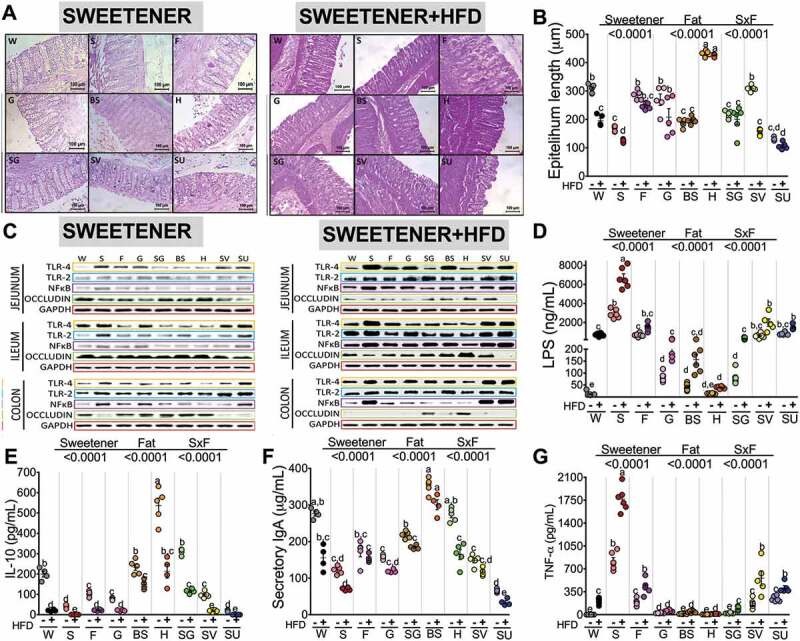
Effect of type of sweetener and the dietary fat content on the epithelium barrier and the inflammatory pathway. (a) Colon histological morphology by hematoxylin-eosin staining, (b) Epithelium layer quantitative analysis, (c) Western blot analysis of TLR4, TLR2, NFκB and occludin in the intestine (d) Serum LPS concentrations, (e) Serum IL-10 concentrations, (f) Serum sIgA concentrations and (g) Serum TNF-α concentrations after the consumption of different types of sweeteners with or without a high-fat diet. The raw data and means±SEMs are shown in each plot, n = 6–7 in each group. W = water, S = sucrose, F = fructose, G = glucose, SG = steviol glycosides, BS = brown sugar, H = honey, SV = steviol glycosides+sucrose and SU = sucralose.

It has been demonstrated that high-fat diets lead to elevated intestinal permeability by modulating the expression of tight junction-associated proteins such as occludin, which suggests that a high-fat diet alters the integrity of the intestinal barrier. Interestingly, the groups fed sucrose and sucralose, particularly exhibited a decrease in epithelial length (Figure 4a,b) and colon occludin abundance (Figure 4c, S2 L); the addition of an HFD to the sweetener, however, significantly reduced the abundance of intestine occludin in almost all groups, especially in the sucrose and sucralose groups (Figure 4d,S2D,h,l). Interestingly, honey maintained epithelium length even after the consumption of an HFD (Figure 4a,b).

Previous evidence has indicated that consumption of an HFD increases serum LPS levels^11^ known as metabolic endotoxemia that has been associated with an increased intestinal permeability. Interestingly, our results showed that serum LPS concentrations increased depending of the type of sweetener and the presence of an HFD. Consumption of an HFD increased serum LPS by 64-fold, whereas sucrose increased serum LPS by 257-fold with respect to the control group. The combination of sucrose+HFD increased serum LPS by 574-fold, indicating that the sweetener significantly contributes to the development of metabolic endotoxemia. Sweeteners such as fructose, SV and sucralose also contributed to a significant increase in metabolic endotoxemia, although not as high as with sucrose. Glucose, steviol glycosides, and brown sugar moderately contributed to an increase in the LPS concentration. Interestingly, honey was the only sweetener that showed similar LPS values as the control group (Figure 4d). For most sweeteners, the addition of an HFD proportionally increased serum LPS in the range of 1 to 2.7-fold. Unexpectedly, the group fed honey+HFD did not develop metabolic endotoxemia. In fact, there was a significant inverse correlation between the circulating concentration of LPS with the abundance of occludin in the intestine (Figure S3A).

TLRs are a family of pattern-recognition receptors that play a critical role in the innate immune system by activating proinflammatory signaling pathways. TLR4 binds to LPS, which in turn triggers a downstream signaling cascade leading to activation of the NF-κB pathway and the transcription of many proinflammatory genes. Since we also observed an increase in the abundance of Gram-positive bacteria after the consumption of sucrose, we measured TLR2, a signaling receptor for another common Gram-positive bacteria-derived peptidoglycan (PGN) and lipoteichoic acid (LTA). We observed that consumption of sucrose, followed by sucralose and SV, significantly increased the abundance of TLR2 in the ileum and colon (Figure 4c,S2f,S2j), which in turn activated NF-κB mainly in the sucrose, sucralose and glucose groups (Figure 4c, S2k). Low concentrations of LPS did not induce TLR4 and did not affect intestinal tight junction permeability through occludin in enterocytes, whereas high concentrations of LPS produced by the consumption of sucrose, sucralose, and SV induced TLR4 and TLR2 abundance (Figure 4c, S2a,e,i) and significantly decreased occludin abundance in the jejunum, ileum and colon (Figure 4c, S2d,h,l). We found a significant positive correlation between serum LPS concentration and TLR4 protein abundance in the intestine (Figure S3B). The increased TLR4 abundance produced by the consumption of sucrose, glucose and sucralose in turn increased the abundance of NF-κB, especially in the colon (Figure 4c, S2K). We found a positive correlation between circulating LPS levels and the relative abundance of NF-κB (Figure S3C). As a consequence, there was a positive correlation between the relative abundance of NF-κB and the relative abundance of TLR4 in the intestine (Figure S3D). The groups fed sucrose+HFD, SV+HFD and sucralose+HFD not only activated TLR4 but also showed TLR2 abundance (Figure 4c, S2b,f,j). In contrast, the consumption of honey, brown sugar and steviol glycosides produced the highest serum IL-10 concentration, an anti-inflammatory cytokine (Figure 4e). One possible explanation for why honey decreased the LPS concentration could in part be due to an increase in secretory immunoglobulin A (sIgA) (figure 4f). sIgA neutralizes LPS in epithelial cells, preventing LPS-induced NF-κB translocation and subsequent proinflammatory responses. In fact, the long-term consumption of honey inhibited the production of proinflammatory cytokines, particularly TNFα (Figure 4g) and induced anti-inflammatory interleukin 10 (IL-10) production (Figure 4e), whereas sucralose significantly reduced sIgA by 76.2% and IL-10 by 99.5% (Figure 4e,f), indicating that honey may help to maintain the gut barrier. We found a significant inverse correlation between sIgA and TNFα (Figure S4A) or LPS levels (Figure S4B) and a positive correlation between sIgA and IL-10 (Figure S4 C); also as expected, as LPS increased, TNFα increased (Figure S4D).

### Consumption of sucrose or sucralose increases short chain fatty acids and GPR43 levels, insulin resistance and glucose intolerance

The consumption of fiber can produce different types of short chain fatty acids (SCFAs) by fermentation by intestinal microbiota that could induce the activation of specific G-protein-coupled receptors (GPCRs) in the intestine. However, it is not well known whether the different sweeteners can produce SCFAs and activate GPR41 and GPR43 to different extents and ultimately enter systemic circulation and activate several metabolic processes.

**Figure 5.**
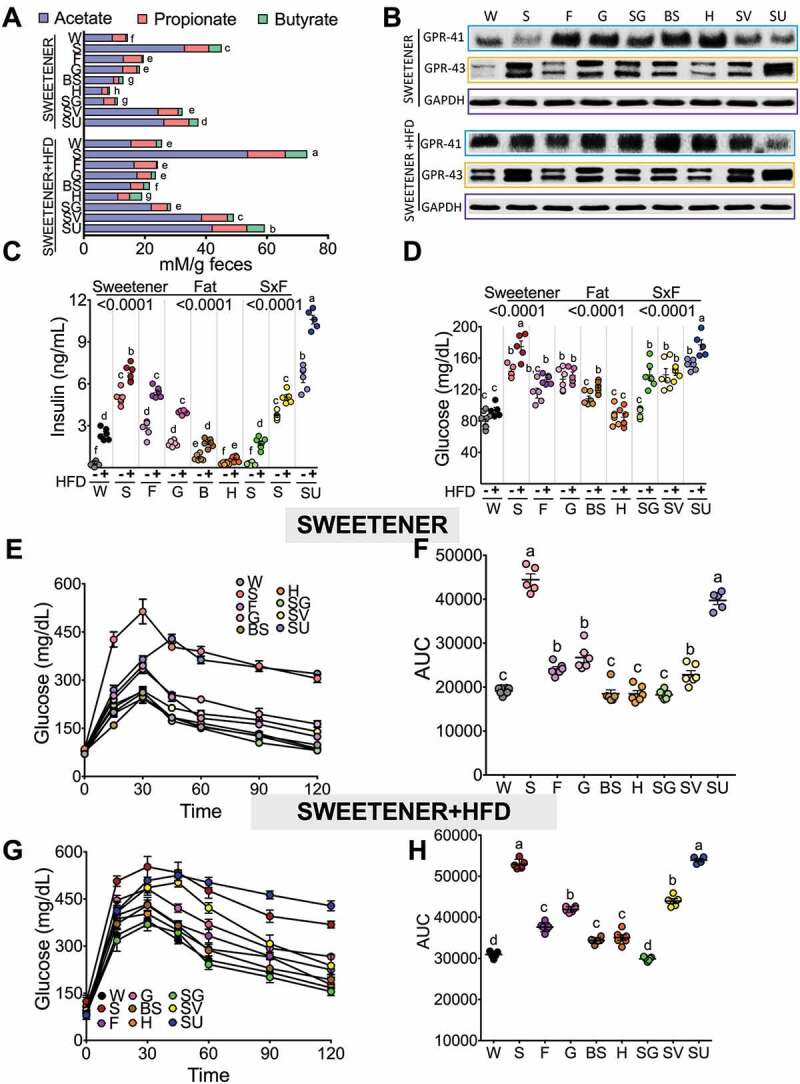
The consumption of different type of sweeteners and fat modifies fecal SCFA production and glucose tolerance. (a) Fecal total SCFAs, (b) Western blot analysis of GPR43 and GPR41 in colon, (c) Serum insulin, (d) Serum glucose concentration, (e, g) glucose tolerance test (ipGTT) and (f, h) Area under the curve after the consumption of different types of sweeteners with or without high-fat diet. The raw data and means±SEMs are shown in each plot, n = 6–7 in each group. W = water, S = sucrose, F = fructose, G = glucose, SG = steviol glycosides, BS = brown sugar, H = honey, SV = steviol glycosides+sucrose and SU = sucralose.

Our data showed that the consumption of sucrose, sucralose and SV were the major producers of total SCFAs (Figure 5a). The addition of an HFD further increased the total SCFA concentration by 78% with respect to the control group. Sucrose+HFD followed by sucralose+HFD and SV+HFD produced the highest concentrations of SCFAs, mainly acetate, which increased by 2.4-, 1.7-, and 1.5-fold with respect to the HFD group (Figure 5a). Interestingly, these groups developed fatty liver associated with an increase in the expression of lipogenic genes, particularly sterol regulatory element binding protein (SREBP-1) and fatty acid synthase (FAS), and an increase in the expression of the gluconeogenic gene phosphoenolpyruvate carboxykinase (PEPCK), particularly in the groups fed sucrose or sucralose (Figure S5A-E), whereas the brown sugar+HFD and honey+HFD groups showed SCFA concentrations similar to those in the C group. The lowest producers of SCFAs were honey, brown sugar and steviol glycosides (Figure 5a).

Interaction between acetate and GPR43 profoundly affects inflammatory responses.^12^ In fact, the highest producers of acetate, the sucrose+HFD and sucralose+HFD groups, were those that showed the highest abundance of GPR43 (Figure 5b, S2 M). All sweeteners+HFD significantly increased the abundance of GPR41 with the exception of sucralose (Figure 5b, S2m,n).

Interestingly, sucrose, sucralose and SV increased the abundance of GPR43 involved in the modulation of insulin secretion, whereas the monosaccharides glucose and fructose as well as brown sugar and honey preferentially induced the abundance of GPR41 (Figure 5b). Interestingly, the group fed sucralose+HFD produced the highest concentration of insulin indicative of severe hyperinsulinemia; it was 43-fold higher than the control group, followed by sucralose and sucrose+HFD at 27- and 28-fold higher, respectively (Figure 5c), whereas those fed honey, even with an HFD, showed a similar insulin concentration to the control group (Figure 5c). Despite the elevation of insulin in the sucralose+HFD and sucrose+HFD groups, they showed elevated fasting serum glucose levels, indicating the development of insulin resistance. The rats fed honey, however, even with an HFD, maintained glucose values similar to the control group (Figure 5d). These results were confirmed with an intraperitoneal glucose tolerance test (ipGTT) showing that groups fed sucrose or sucralose with and without an HFD showed glucose intolerance (Figure 5e-h). Interestingly, the results showed a positive correlation between area under the curve for glucose after an ipGTT and the relative abundance of GPR-43 (Figure S6). One of the limitations of an ipGTT is the absence of an incretin effect with intraperitoneal injections. Not surprisingly, the glucose and insulin levels after an oral GTT are lower than those seen with the intraperitoneal route, although the area under the curve for glucose positively correlates with GPR43 protein abundance.

### Differences in metabolic endotoxemia due to the type of sweetener and high-fat diet were associated with changes in energy expenditure, metabolic inflexibility and fatty acid oxidation

**Figure 6.**
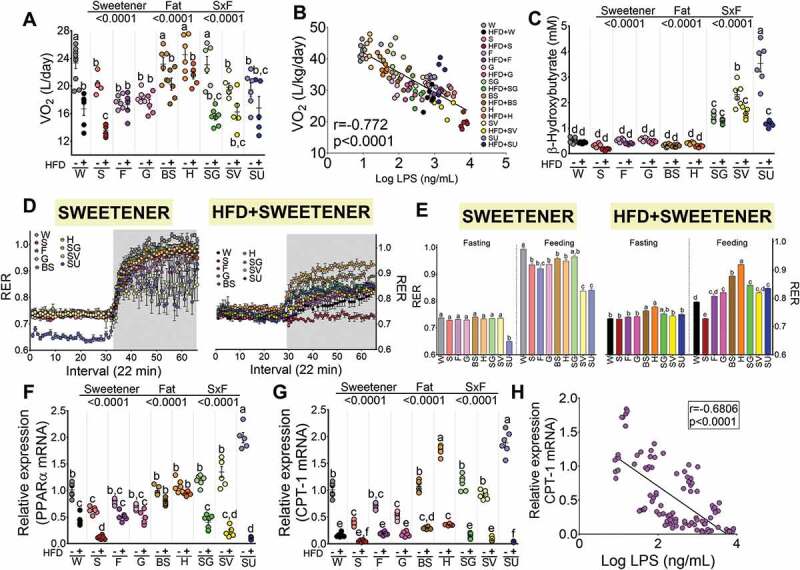
Energy expenditure and fatty acid oxidation depends on the type of sweetener and the high-fat diet. (a) O_2_ consumption (VO_2_ expressed as L/day corrected for body mass by ANCOVA), (b) Correlation between oxygen consumption and serum LPS levels, (c) Liver PPARα gene expression, (d) Liver CPT-1 gene expression, (e) Correlation between CPT-1 expression and serum LPS concentration, (f) Serum β-hydroxybutyrate concentration after the consumption of different types of sweeteners with or without high-fat diet, (g, h) Respiratory exchange ratio (RER) after the consumption of different types of sweeteners with or without a high-fat diet. Data are shown as the mean±SEM, n = 6–7 in each group. W = water, S = sucrose, F = fructose, G = glucose, SG = steviol glycosides, BS = brown sugar, H = honey, SV = steviol glycosides+sucrose and SU = sucralose.

As the sweeteners exerted differential effects on weight gain, body composition and metabolic endotoxemia, we further studied energy expenditure by indirect calorimetry. The results showed that the groups fed steviol glycosides and honey had the highest VO_2_ consumption and energy expenditure, whereas the addition of high fat to the diet decreased the VO_2_ consumption, particularly in the groups fed sucrose+HFD and steviol glycosides+HFD. It is noteworthy that the groups fed honey and brown sugar, despite the addition of an HFD, maintained both an elevated VO_2_ consumption (Figure 6a) and energy expenditure (Figure S10).

We therefore assessed whether the VO_2_ consumption was associated with the circulating concentration of LPS. Interestingly, we observed a significant inverse correlation between the VO_2_ consumption and the level of LPS, where groups fed honey, even in the presence of an HFD, maintained low circulating concentrations of LPS and had high VO_2_ consumption, while the group fed sucrose+HFD had the highest concentration of circulating LPS and the lowest VO_2_ consumption (Figure 6b). We could not determine whether a specific genus or species was associated with the increase in VO_2_ consumption, although we did observe that a higher abundance of *B. fragilis* in the sucralose+HFD group was associated with low VO_2_ compared to the control group (Figure S7). The increase in energy expenditure is associated with an increase in the use of energy substrates. We found that some sweeteners modified the expression of the transcription factor Peroxisome Proliferator-activated receptor α (PPARα) and the enzyme carnitine palmitoyl transferase-1 (CPT-1), which are involved in the oxidation of fatty acids in the mitochondria (Figure 6c,d). It is notable that sucralose was more capable of stimulating the expression of PPARα and CPT-1 than any other sweetener (Figure 6c,d). Our results also revealed that consumption of honey, brown sugar and steviol glycosides significantly stimulated the relative abundance of the mRNA for these proteins in the liver, indicating that the consumption of these sweeteners can stimulate VO_2_, and therefore energy expenditure, by an increase in fatty acid oxidation. The addition of a high-fat diet to most of the sweeteners, however, decreased the expression of these genes (Figure 6c,d). In fact, we observed a significant inverse correlation between the relative abundance of hepatic CPT-1 with the circulating concentration of LPS (Figure 6e).

Thus, we assessed the respiratory exchange ratio (RER) in all of the groups to evaluate the type of energy substrate used for energy expenditure. A low RER (0.70) reflects predominantly fat oxidation, whereas a high RER (1.00) is indicative of glucose oxidation. The results showed two findings; first, with most natural sweeteners, there was a switch in RER from 0.7 in the fasting state to 1 in the fed state, indicating a metabolic flexibility in the use of energy substrates (Figure 6g,h). However, in the group fed SV, the RER changed from 0.7 during the fasting state to 0.84 after feeding, indicative of partial metabolic inflexibility. We were surprised by the response produced by the group fed sucralose, since during the fasting state the RER was around 0.65, and after feeding, the RER was 0.83 (Figure 6g,h). These results indicated that sucralose generates metabolic inflexibility in the use of energy substrates. They also showed that during fasting, an RER below 0.7 suggested a ketogenic state; there was the significant elevation in circulating concentrations of β-hydroxybutyrate (figure 6f), the most abundant of the ketone bodies, which was accompanied by an increase in the circulating concentrations of fatty acids due to an increase in the phosphorylation of the hormone sensitive lipase (HSL) in adipose tissue (Figure S8).

## Discussion

Sugar consumption often exceeds the recommended level of 10% of the total energy intake in children and adults,^13^ and the excess sugar in foods or sweetened beverages and noncaloric sweeteners have been associated with glucose intolerance;^5^ high postprandial insulin peaks;^14^ and an increased risk of metabolic syndrome, diabetes,^15^ obesity,^16^ and cardiovascular disease, among others. According to the Dietary Guidelines for Americans 2015–2020, the average consumption of added sugars is approximately 13% of the energy intake; in some groups, including children, adolescents, and young adults, however, the consumption reaches approximately 17%.^17^ This value is close to the amounts of caloric sweeteners provided in the rats’ experimental diets in our study. In addition, approximately 47% of the added sugar intake in the American population has been described as coming from beverages, which partially resembles our study design for providing the sweetener in drinking water. It has been postulated that the link between sweeteners and glucose intolerance occurs in part through the gut microbiota by creating distinct environments for microbes. The pattern of response of the microbes to specific sweeteners varies depending in part on how each sweetener is absorbed and transported in the small intestine and colon and the capacity to regulate the release of incretins (Sanchez-Tapia et al., 2019). It is currently unknown how changes in the gut microbiota due to the consumption of different sweeteners modulate metabolic endotoxemia, the production of SCFAs, body composition and energy expenditure, among other metabolic consequences.

Our results clearly established that the effects of caloric and NCS differentially modify weight gain and body composition despite the consumption of a high-fat diet. The groups that consumed sucrose and sucrose+HFD had the highest body weight gain, % body fat and the lowest lean mass, whereas sucralose intake resulted in an increase in % body fat, although it is an NCS, probably due to the highest total intake of all of groups (Figure S9); of note, however, this group showed the highest ketone body formation. It has been demonstrated that drinking several artificial sweeteners increases hunger ratings,^18^ and in fact in our study, the group fed sucralose showed the highest total energy intake and the highest levels of ketone bodies. This result may be because sucralose was the sweetener that most stimulated the expression of the transcription factor PPARα, which is involved in fatty acid oxidation, ketone body formation, and gluconeogenesis.^19^ This may explain why the consumption of sucralose considerably increased the formation of ketone bodies and gluconeogenesis, increasing glucose and insulin levels and producing glucose intolerance of the same magnitude as that seen with sucrose.

On the other hand, less refined caloric sweeteners, particularly honey and brown sugar, showed less of an effect on body weight and body fat, which was consistent with previous results that demonstrated that consumption of these sweeteners led to small adipocyte size, an increase in adiponectin gene expression in white adipose tissue, and an increase in uncoupling protein-1 (UCP-1) in brown adipose tissue indicative of functional adipocytes.^20^

Surprisingly, not all sweeteners increased circulating levels of LPS to the same extent. In particular, the groups consuming sucrose showed the highest LPS concentrations, while those consuming honey or brown sugar had the lowest LPS levels, even though all three sweeteners provide similar Kcal/g. More remarkably in our study, the LPS elevation from consumption of a high-fat diet depended on the type of sweetener consumed, with the combination of sucrose+HFD raising the LPS concentration the most. However, the group that consumed sucralose had the highest total energy intake, although sucralose is a noncaloric sweetener, but it also showed high concentrations of LPS, indicating that the metabolic endotoxemia was not exclusively related to total energy intake but also to the type of sweetener consumed. The most striking result is that the group fed honey+HFD, despite energy consumption similar to that of the sucrose+HFD group, showed similar LPS concentrations to the control group. These results could be partially explained by the fact that honey contains several monosaccharides, a low concentration of sucrose of approximately 1–2%, and bioactive compounds, particularly polyphenols with antioxidant activity (Figure S1).

Our results suggest that the interaction an HFD and the type of sweetener could selectively modify the gut microbiota. In fact, in our study, the greatest diversity of gut microbiota was seen with natural sweeteners, and the lowest diversity was observed with NCS with and without the consumption of an HFD. The changes in α-diversity were accompanied by changes in gene richness of the gut microbiota according to metagenomic analysis. In particular, those rats fed honey and brown sugar had the highest gene richness, while the groups fed an HFD showed a reduction in both α-diversity and gene richness, especially in those rats fed NCS such as sucralose and steviol glycosides. These results are consistent with those of previous studies in both rodents and humans that have demonstrated that the use of NCS generates gut microbiota dysbiosis.^5,21,22^

It is important to point out that the ratio of the main phyla Bacteroidetes/Firmicutes was similar in most of the sweeteners, with the exception of steviol glycosides and sucralose. However, the impacts of the NCS SV and sucralose at the genus level were the reduction of *Lactococcus, Mucispirillum* and *Bidifobacterium*, most of which are involved in maintaining host gut health.^23–25^ Consumption of honey, however, increased the *Bifidobacterium* genus, and at the species level, it increased *Faecalibacterium prausnitzii, Butyricoccus pullicaecorum* and *Bifidobecaterium animalis*, which are associated with gut health and metabolic benefits.^8,9,26^ Interestingly, based on our metagenomic analysis, we observed that the consumption of an HFD increased the number of genes involved in LPS synthesis, especially in the groups fed steviol glycosides, sucralose and sucrose. In contrast, it should be noted that the groups fed honey, brown sugar or steviol glycosides had the lowest number of genes involved in the production of LPS and had the lowest circulating levels of LPS, even in the group fed honey+HFD. These results could depend on the composition of these sweeteners. Brown sugar is a natural sweetener that contains 5 to 6.5% molasses, although there is scarce indirect information that molasses may modulate the gut microbiota.^27^ Molasses contains a significant amount of metal ions that can contribute to the functionality of some species present in the intestinal microbiota, although there are still no studies that have demonstrated this effect. Honey contains an important number of polyphenols^28^ that could potentially modify the intestinal microbiota to produce beneficial metabolic effects. Interestingly, honey contains several monosaccharides and very low concentrations of sucrose, although more studies are still necessary to prove these effects.

Changes in circulating levels of LPS are associated with changes in both the structure of the intestinal epithelial mucosa and the expression of markers of the inflammatory response. The pro-inflammatory effect observed from the consumption of a high-fat diet was only seen in the sucrose, SV or sucralose groups, where an increase in the expression of TLR4, TNFα and NF-κB was observed. TLR4 expressed in enterocytes is recognized by LPS and represents one of the most powerful indicators of microbial inflammation. Interestingly, mice lacking TLR4 are protected against high-fat diet-induced insulin resistance, suggesting that TLR4 is a molecular link between nutrition and inflammation.^29^ The interaction between LPS and TLR4 induces the synthesis of proinflammatory cytokines, such as TNFα, which in turn work as endogenous inflammatory mediators by interacting with receptors found in different target cells. Following ligand binding, TLR4 dimerizes with MyD88 and triggers the NF-κB pathway, activating the transcription of genes of pro-inflammatory cytokines and participating in inflammasome regulation. Recent studies have revealed an alternative, TLR4-independent activation of pro-inflammatory responses to LPS. When high concentrations of LPS persist, LPS can be aberrantly found in the cytoplasm of macrophages, where it binds to murine caspase-11 and activates a noncanonical inflammasome leading to the generation of pro-inflammatory IL-1β,^30^ an effect that deserves further study. However, in the present work, we observed a positive association between LPS with TLR-4 and NF-κB and a negative association with occludin abundance, suggesting that an increase in LPS mainly stimulates NF-κB via TLR4. In contrast, the consumption of honey, brown sugar and steviol glycosides not only decreased the expression of these inflammation markers, but honey in particular increased the expression of anti-inflammatory cytokines such as IL-10 in addition to increasing the concentration of sIgA.

The metagenomic analysis revealed that the type of sweetener also modified the number of genes involved in SCFA synthesis in the gut microbiota. Evidence suggests that fiber^31^ is the main substrate for the production of SCFAs by gut microbiota, and we also found that the type of sweetener can specifically modify the production of SCFAs. Some SCFA effects are mediated via the GPR41 and GPR43 receptors present in the enteroendocrine L cells in the intestines,^32,33^ mediating protective immunity and inflammation.^6^ Our results showed that sucrose, SV and sucralose were the major producers of acetate, and the addition of an HFD significantly increased the production of acetate that was associated with the development of hepatic steatosis. Some evidence suggests that SCFAs promote gluconeogenesis and lipogenesis,^34^ which is in agreement with our results. High concentrations of acetate found in the sucrose+HFD and sucralose+HFD groups were associated with an increase in the abundance of GPR43 and the development of glucose intolerance and insulin resistance, whereas those rats fed honey+HFD showed an opposite pattern similar to the control group.

On the other hand, there is evidence that LPS infusion mediates the development of obesity, increasing the amount of adipose tissue and glucose intolerance via activation of TLR4 in adipocytes.^35^ This evidence suggests that changes in LPS levels could be associated with changes in fat mass and VO_2_ consumption. In fact, we found that the groups fed honey or brown sugar with low levels of LPS had the highest VO_2_ consumption and the lowest body fat, where those groups fed sucrose, SV or sucralose showed the opposite pattern. Low circulating levels of LPS were also associated with an increase in fatty acid oxidation, leading to higher energy expenditure and greater metabolic flexibility in the use of energy substrates.^36^

The increased prevalence of obesity has become a major health problem worldwide and has been associated with the type of sweetener consumed in addition to the presence of saturated fat. Although many studies have examined the benefits and drawbacks of sweeteners, little information is available comparing the different types of sweeteners. Based on the results of the present study, we were able to demonstrate for the first time how different caloric and noncaloric sweeteners can determine the presence or absence of metabolic endotoxemia and hence the adverse effects on the metabolism of carbohydrates and lipids due to selective modulation of the gut microbiota and the production of short-chain fatty acids. However, further studies should assess the long-term effects of sweeteners such as honey in humans with an appropriate intervention duration, comparator, and outcomes.

## Materials and methods

### Animals

Male Wistar rats aged 5 weeks were obtained from the National Institute of Medical Sciences and Nutrition. The animals were housed in individual cages and maintained at a controlled room temperature with 12-h light-dark cycles and free access to water and food. Rats were divided into 18 groups; 9 groups were fed a control diet (C) according to the recommendations of the American Institute of Nutrition^7^ and different sweeteners in their drinking water (n = 6 per group) for 4 months. The other 9 groups were fed a high-fat diet (HFD) and different sweeteners in their drinking water for 4 months (n = 6 per group) Table S1. At the end of study, the rats were fasted for 10 h and then sacrificed under general anesthesia with sevoflurane. Tissues were rapidly removed and stored at −70ºC, and serum was obtained by centrifugation of blood at 1500x*g* for 10 min and stored at −70ºC. The Animal Care and Use Committee (CICUAL) of the National Institute of Medical Sciences and Nutrition, Mexico City (CICUAL-1735) approved the protocol.

To calculate the sample size, the formula for the comparison of means was used:

$$n=\frac{2s^{2}{\left(Z\alpha +Z\beta \right)}^{2}}{\Delta^{2}}$$

Where: n = sample size; s = standard deviation; Zα = Type I error (confidence level α = 0.05 corresponding to a value of Z = 1.96); Zβ = with a power of 80% (value of Z = 0.84); Δ = difference in magnitude between means of the treatments (amplitude). The work of Suez^5^ was used as a reference to calculate the sample size. After applying the formula with the glucose tolerance, weight, and intestinal microbiota data, we obtained n = 5, 6 and 9. To reduce the number of animals, a sample size of 6 Wistar rats was chosen for each experimental group in this project.

### Food intake

A known amount of food was placed in the feeder, and the amount of remaining food was measured 48 h later. The amount of food consumed was calculated by the difference divided by 2 days and expressed as food intake per rat per day. Food intake was monitored throughout the entire study.

### Body weight gain

Every other day, rats were weighted. Body weight gain was calculated by difference with respect to the initial body weight.

### Water intake with sweeteners

All of the sweeteners were dissolved in water to a concentration of 10%, with the exception of steviol glycosides (SG) and sucralose (SU), for which the concentrations were 2.5% and 1.5%, respectively.

### Ingredients in the diets

Steviol glycosides, sucralose, sucrose+steviol glycosides (SV) and brown sugar were donated by Metco, S.A de C.V. Sucrose was obtained from Zucarmex, S.A. de C.V., México; orange blossom honey was from Alimentos Finisterre, S de R. L de C.V. Tequisquiapan, Qro, México; glucose was from Drogueria Cosmopolita, S.A de C.V, México; and fructose was from Savien Frusweet S.A de C.V., México. Casein and the mineral and vitamin mixes were obtained from Teklad Test Diets, Madison, WI, USA.

### Biochemical parameters

Serum insulin, TNFα (Alpco Diagnostics, Salem, NH, USA), LPS (Cloud-Clone Corp. Texas, USA), sIgA (My BioSource, San Diego, CA, USA), and IL-10 (R&D Systems, Inc., Minneapolis, MN, USA) were measured using commercial ELISA kits. Serum triglycerides, glucose, and total and LDL cholesterol were determined by enzymatic colorimetric assays using a COBAS C11 autoanalyzer (Roche, Basel, Switzerland).

### Intraperitoneal glucose tolerance test

The intraperitoneal glucose tolerance test was determined as previously described^37^ administering an intraperitoneal glucose injection according to NIH recommendations^38^ of 2 g per kg body weight in rats after an 8-hour fast. Blood samples were collected from the tail vein at 0, 15, 30, 45, 60, 90, and 120 min after administration of the glucose. Blood glucose concentration was measured using a FreeStyle Optium glucometer (Abbot Laboratories, Abbot Park, IL, USA). The area under the curve was determined by the trapezoid method.

### Energy expenditure by indirect calorimetry

Rats were placed in a noninvasive in vivo calorimetric chamber of an Oxymax open circuit indirect calorimeter (Oxymax, Columbus Instruments, OH, USA) with either a control diet or a high-fat diet and water (ad libitum) to assess energy expenditure using measurements of oxygen consumption and carbon dioxide production over 24 h. The rats were previously adapted to the chambers for at least 12 h. From the measurements of VO_2_ and VCO_2_, the respiratory exchange ratio (RER) can be calculated to assess energy fuel utilization and energy expenditure.^39^ The RER is the ratio between the amount of CO_2_ produced via metabolism and the oxygen used to indicate which fuel has been metabolized to supply the body with energy. We assessed the respiratory exchange ratio (RER) in all groups to evaluate the type of energy substrate used for energy expenditure. A low RER (0.70) reflects predominantly fat oxidation, whereas a high RER (1.00) is indicative of glucose oxidation. Energy expenditure data were divided by body weight, and the results were presented by plotting individual data and analyzing body-weight effect using analysis of covariance (ANCOVA).^40^

### Evaluation of whole body composition

Rats were placed into a thin-walled plastic cylinder with a cylindrical plastic insert added to limit movement within a quantitative magnetic resonance imaging system (Echo MRI, Houston, Tx, USA). While in the tube, the animals were briefly subjected to a low-intensity (0.05 Tesla) electromagnetic field to measure fat and lean mass.^41^ Fat and lean mass were monitored every 2 weeks.

### Liver gene expression

Liver total RNA was extracted using TRIzol following the manufacturer’s instructions, and the mRNA was quantified using a Qubit 3.0® fluorometer (Invitrogen, Pittsburg, USA). mRNA abundance was measured by real-time quantitative PCR using SYBR® Green assays (Roche), using HPRT and cyclophilin as references for normalization using the Light Cycler 480 system (Roche Applied Science, Indianapolis, IN, USA). All samples were checked for quality and had an A260/A280 ratio of 1.8–2.0. All primer sequences were blasted to confirm matches to intended gene targets. All samples were run in triplicate.

### Protein abundance

At the end of the experiment, intestine and adipose tissue samples were collected from the rats after 10 h of fasting. The total protein of different sections of intestine (n = 6) was extracted and quantified using the Bradford assay (Bio-Rad, Hercules, CA, USA) and stored at −70ºC. Protein samples were separated on 10% acrylamide gels with 30 µg protein per lane and blotted onto PVDF membranes. Membranes were blocked in a blocking buffer consisting of 3% BSA in TBS Tween for 60 min at room temperature and incubated overnight at 4ºC with the primary antibody and the following primary and secondary antibodies in the blocking buffer: anti-pHSL (Cell Signaling, 4139, rabbit polyclonal IgG) at 1:1000; anti-TLR4 (Santa Cruz, SC-293072, mouse monoclonal IgG) at 1:1000; anti-TLR2 (Santa Cruz, SC-10739, rabbit polyclonal IgG) at 1:1000; anti-NFKB (Santa Cruz, SC-372, rabbit polyclonal) at 1:1500; anti-occludin (Abcam, AB167161, rabbit monoclonal IgG) at 1:100,000; anti-GPR41 (LSBio, LS-C357088-100, rabbit polyclonal) at 1:1500; and anti-GPR43 (Santa Cruz, SC-32906, rabbit polyclonal) at 1:1500. The blots were incubated with anti-rabbit (Abcam, AB6885) or anti-mouse (Abcam, AB6789) secondary antibodies conjugated with horseradish peroxidase (1:15000). GAPDH (1:3500) was used to normalize the data. Images were analyzed with ChemiDoc^TM^ XRS + System Image Lab^TM^ software (Bio-Rad, Hercules, CA, USA). The assays were performed three times using independent blots.

### Fecal DNA extraction and 16S rRNA sequencing

A fecal sample from all animals was collected after 4 months of the treatment with different sweeteners. Fecal samples were frozen at −80°C until analysis. DNA extraction was performed using a QIAamp DNA Stool Mini Kit (Qiagen, U.S.A.) according to the manufacturer’s instructions. Variable regions 3–4 of the 16S rRNA gene were amplified using specific forward (5ʹ TCGTCGGCAGCGTCAGATGTGTATAAGAGACAGCCTACGGGNGGCWGCAG 3ʹ) and reverse primers (5ʹ GTCTCGTGGGCTCGGAGATGTGTATAAGAGACAGGACTACHVGGGTATCTAATCC 3ʹ) containing the Illumina adapter overhang nucleotide sequences. Ampure XP bits were used to purify the 16S V3-V4 amplicons, which were quantified by high resolution capillary electrophoresis (QIAGEN, Germany) The amplicon size was approximately 550 bp. An index PCR was then carried out to attach dual indices using a Nextera XT v2 Kit. The amplicon size was approximately 610 bp, and the concentration of double-stranded DNA was measured using a fluorometer Qubit 3.0 with a high-sensitivity kit. The final amplicon library was pooled in equimolar concentrations. Sequencing was performed on the Illumina MiSeq platform (MiSeq Reagent Kit V.3, 600 cycles) at 15 pM with 20% Phyx infection according to the manufacturer’s instructions to generate paired-end reads of 300 bases in length in each direction.

### Bioinformatic analysis

For taxonomic composition analysis, Custom C# and python scripts in the Quantitative Insights Into Microbial Ecology (QIIME) software pipeline 1.9 were used to process the sequencing files.^42^ The sequence outputs were filtered for low-quality sequences (defined as any sequences that are <200 bp or >620 bp, sequences with any nucleotide mismatches to either the barcode or the primer, sequences with an average quality score of <30, and sequences with ambiguous bases >0). Sequences were checked for chimaeras with Gold.fa, and chimeric sequences were filtered out. The analysis started by clustering sequences within a percent sequence similarity into operational taxonomic units (OTUs). Ninety-four percent of the sequences passed filtering, resulting in 54144 ± 23931 sequences/sample on average with a 97% similarity threshold. OTU picking was performed with the tool set from QIIME, using the Usearch method.^43^ OTUs were picked against the GreenGenes v.13.9 database. Ninety-seven percent of the OTUs were selected from the database. After the resulting OTU files were merged into one overall table, taxonomy was assigned based upon the GreenGenes reference taxonomy database. Thus, 99.8%, 99.8%, 99.4%, 92.3%, 80.16% and 39.21% of the reads were assigned to the phylum, class, order, family, genus and species level, respectively. Species richness (Observed, Chao1) and alpha diversity measurements (Shannon) were calculated, and we estimated the within-sample diversity at a rarefaction depth of >19011 reads per sample. Weighted and unweighted UniFrac distances were used to perform the principal coordinate analysis (PCoA). Microbial sequence data were pooled for OTU comparison and taxonomic abundance analysis but separated by batch in the principal coordinate analysis (PCoA) to obtain clear PCoA figures. For even sampling, a depth of 19011 sequences/sample was used. PCoAs were produced using Emperor, and community diversity was determined by the number of OTUs and beta diversity, measured by unweighted and weighted UniFrac distance matrices in QIIME. ANOSIM, a permutational multivariate analysis of variance, was used to determine statistically significant clustering of groups based upon microbiota structure distances. To determine the difference in the OTU relative abundance of genera and species among the different groups, a heatmap at the species or genus level was performed using the script of QIIME “otu_category_significance.py”, which uses an ANOVA to determine whether OTU relative abundance is different between different treatments; finally, we chose the 10 with the most significance.

### Shotgun metagenomics sequencing analysis

A fecal sample was collected from all animals after 4 months of the treatment with different sweeteners with and without a high-fat diet. Fecal samples were frozen at −80°C. DNA extraction was carried out using a QIAamp DNA Stool Mini Kit (Qiagen, U.S.A.) according to the manufacturer’s instructions. We tagmented genomic DNA (gDNA) with a Nextera XT kit. We used the Nextera transposome to tagment gDNA, which is a process that fragments and tags DNA with adapter sequences. Ampure XP bits were used to purify the libraries, and subsequently, the libraries were evaluated in the integrity and the size of the fragments quantified on Qiaxcel (QIAGEN, Germany). The library size distribution was between 250–1000 bp, and the library was amplified using an IDT kit for Illumina Nextera UD Indexes. The concentration of double-stranded DNA was measured using a fluorometer Qubit 3.0 with a high-sensitivity kit. The final normalized library sequencing was performed on the Illumina HiSeq 2500 platform (HiSeq Rapid Cluster Kit v2) at 11 pM with 1% of Phix.

### Statistical analysis

Statistical analyses were performed using GraphPad Prism v7.0 using two-way ANOVA followed by a post hoc Tukey test. Data are presented as the means ± SEMs. Differences between group means were considered significant at *p* < .05. Sample sizes can be found in the figure legends, where n represents the number of animals used in the experiment.

## Supplementary Material

Supplemental Material
