# Supplementary material for: The development of metabolic endotoxemia is dependent on the type of sweetener and the presence of saturated fat in the diet
Source: Gut Microbes. 2020 Aug 17;12(1):1801301. doi: 10.1080/19490976.2020.1801301 (PMC7524302; doi:10.1080/19490976.2020.1801301)
Supplement: Supplemental Material [file KGMI_A_1801301_SM9876.zip › Supplementary information/supplementary tables S1, S2, S3.docx]

| Table S1. Composition of purified diets used in this study | | |
| --- | --- | --- |
| Ingredient | AIN-93 Diet | High Fat Diet (HFD) |
| Cornstarch | 39.775 | 23.903 |
| Casein (>85% protein) | 20.000 | 24.000 |
| Dextrinized cornstarch | 13.200 | 10.267 |
| Sucrose | 10.000 | 7.778 |
| Soybean oil | 7.000 | 7.000 |
| Fiber Cellulose | 5.000 | 5.000 |
| Mineral Mix (AIN-93G-MX) | 3.500 | 3.500 |
| Vitamin Mix (AIN-93-VX) | 1.000 | 1.000 |
| L-Cystine | 0.300 | 0.300 |
| Choline bitartrate (41.1% choline) | 0.2500 | 0.2500 |
| Tert-butylhydroquinone | 0.0014 | 0.0014 |
| Lard | - | 17 |

* The composition of the diets is given in g/100g diet

| Table S2. Pairwise ADONIS metagenomic analysis | | | |
| --- | --- | --- | --- |
| Pairs | F.Model | R^2^ | p Value |
| Overall | 9.7441 | 0.27647 | 0.001 |
| Control vs Caloric Sweeteners | 1.736257 | 0.03562556 | 0.043 |
| Control vs HFD + Caloric Sweeteners | 3.700303 | 0.08278026 | 0.001 |
| Control + HFD + NAS | 7.146241 | 0.33794379 | 0.001 |
| Control vs NAS | 9.103 | 0.34873385 | 0.001 |
| Caloric Sweeteners vs HFD + Caloric Sweeteners | 12.827317 | 0.14440734 | 0.001 |
| Caloric Sweeteners vs HFD + NAS | 12.545445 | 0.20384035 | 0.001 |
| Caloric Sweeteners vs NAS | 14.059499 | 0.21283084 | 0.001 |
| HFD + Caloric Sweeteners vs HFD +NAS | 6.769657 | 0.13601976 | 0.001 |
| HFD + Caloric Sweeteners vs NAS | 13.684103 | 0.22927551 | 0.001 |
| HFD + NAS vs NAS | 7.877171 | 0.29308036 | 0.001 |

| Table S3. Pairwise ADONIS metagenomic analysis by individual groups | | | |
| --- | --- | --- | --- |
| Pairs | F.Model | R2 | P Value |
| Overall | 6.5696 | 0.55652 | 0.001 |
| C vs G | 3.207712 | 0.2257726 | 0.001 |
| C vs SG | 5.770348 | 0.3440804 | 0.002 |
| C vs HFD+F | 3.868046 | 0.2601583 | 0.002 |
| C vs HFD+G | 4.460601 | 0.2885141 | 0.001 |
| C vs HFD+SG | 11.968585 | 0.4993447 | 0.001 |
| C vs HFD+H | 3.579265 | 0.2455038 | 0.002 |
| C vs BS | 3.01178 | 0.2149463 | 0.001 |
| C vs H | 2.945567 | 0.2112189 | 0.002 |
| C vs SU | 8.335126 | 0.4310872 | 0.001 |
| C vs SV | 9.208233 | 0.4556674 | 0.002 |
| F vs HFD+G | 2.944042 | 0.2274438 | 0.002 |
| F vs HFD+SG | 10.235001 | 0.4819873 | 0.001 |
| F vs HFD+H | 2.154827 | 0.1772816 | 0.002 |
| F vs BS | 4.120981 | 0.2918339 | 0.002 |
| F vs S | 3.28999 | 0.247554 | 0.002 |
| F vs SU | 7.913109 | 0.4417496 | 0.002 |
| G vs SG | 8.153043 | 0.4491282 | 0.001 |
| G vs HFD+SG | 19.574938 | 0.6402282 | 0.001 |
| G vs HFD+H | 9.34962 | 0.483194 | 0.001 |
| G vs HFD+S | 8.005522 | 0.4446148 | 0.002 |
| G vs HFD+SU | 17.227084 | 0.6828805 | 0.002 |
| G vs HFD+SV | 13.186377 | 0.5943457 | 0.001 |
| G vs BS | 2.847059 | 0.2216117 | 0.002 |
| G vs H | 2.692047 | 0.212105 | 0.002 |
| G vs S | 3.945657 | 0.2829309 | 0.001 |
| G vs SV | 14.961009 | 0.5993752 | 0.001 |
| SG vs HFD+G | 9.02945 | 0.4744987 | 0.002 |
| SG vs HFD+SG | 14.324614 | 0.56564 | 0.002 |
| SG vs BS | 8.744392 | 0.4665071 | 0.002 |
| SG vs SV | 7.542746 | 0.4299638 | 0.002 |
| HFD vs HFD+SG | 11.718268 | 0.5158082 | 0.001 |
| HFD vs HFD+S | 2.788933 | 0.2180739 | 0.001 |
| HFD vs SU | 11.107023 | 0.526224 | 0.002 |
| HFD vs SV | 11.828421 | 0.5418817 | 0.002 |
| HFD+F vs HFD+G | 4.332378 | 0.3022791 | 0.001 |
| HFD+F vs HFD+SG | 14.732921 | 0.572532 | 0.001 |
| HFD+F vs HFD+BS | 3.399672 | 0.2741743 | 0.002 |
| HFD+F vs BS | 11.468781 | 0.5342074 | 0.002 |
| HFD+F vs H | 10.174067 | 0.5043141 | 0.002 |
| HFD+G vs HFD+SG | 12.717358 | 0.5362047 | 0.002 |
| HFD+G vs HFD+H | 3.640866 | 0.2669087 | 0.001 |
| HFD+G vs SU | 11.950283 | 0.544425 | 0.002 |
| HFD+G vs SV | 12.80231 | 0.5614479 | 0.002 |
| HFD+SG vs HFD+BS | 13.371739 | 0.5721328 | 0.002 |
| HFD+SG vs HFD+H | 13.959996 | 0.5592948 | 0.001 |
| HFD+SG vs HFD+S | 14.841335 | 0.5743254 | 0.001 |
| HFD+SG vs HFD+SV | 3.604973 | 0.2649747 | 0.002 |
| HFD+SG vs BS | 21.719839 | 0.6638125 | 0.002 |
| HFD+SG vs H | 23.234373 | 0.6786855 | 0.001 |
| HFD+SG vs S | 21.850343 | 0.6651481 | 0.002 |
| HFD+SG vs SU | 16.698565 | 0.6028675 | 0.001 |
| HFD+SG vs SV | 16.732828 | 0.6033582 | 0.002 |
| HFD+BS vs HFD+H | 2.871733 | 0.2418967 | 0.002 |
| HFD+BS vs BS | 7.360089 | 0.4498807 | 0.002 |
| HFD+BS vs S | 6.378172 | 0.4147549 | 0.002 |
| HFD+H vs BS | 10.831253 | 0.5199521 | 0.002 |
| HFD+H vs H | 9.722976 | 0.4929771 | 0.002 |
| HFD+H vs SU | 12.599204 | 0.5575065 | 0.001 |
| HFD+S vs HFD+SV | 10.004529 | 0.5264287 | 0.002 |
| HFD+S vs BS | 9.734913 | 0.4932838 | 0.001 |
| HFD+S vs H | 8.866674 | 0.4699649 | 0.002 |
| HFD+S vs SU | 13.108301 | 0.5672551 | 0.001 |
| HFD+SV vs SV | 9.263534 | 0.5072148 | 0.002 |
| BS vs S | 4.296547 | 0.3005304 | 0.001 |
| H vs S | 3.775443 | 0.2740705 | 0.002 |
| H vs SV | 18.297892 | 0.6466168 | 0.001 |
| S vs SU | 14.209543 | 0.5869397 | 0.002 |
| C vs HFD | 3.104905 | 0.2201294 | 0.003 |
| C vs HFD+S | 3.338193 | 0.2328183 | 0.003 |
| C vs S | 2.392196 | 0.1786261 | 0.003 |
| F vs G | 3.474244 | 0.2578433 | 0.003 |
| F vs SG | 5.448323 | 0.3526806 | 0.003 |
| F vs HFD+F | 2.536868 | 0.2023526 | 0.003 |
| F vs HFD+SV | 6.421437 | 0.4163968 | 0.003 |
| G vs HFD | 6.255592 | 0.3848271 | 0.003 |
| G vs HFD+F | 10.072604 | 0.5018085 | 0.003 |
| G vs HFD+G | 10.717038 | 0.5173055 | 0.003 |
| G vs SU | 13.313005 | 0.5710549 | 0.003 |
| SG vs HFD | 8.616659 | 0.4628467 | 0.003 |
| SG vs H | 9.034167 | 0.474629 | 0.003 |
| SG vs SU | 8.162832 | 0.4494251 | 0.003 |
| HFD vs HFD+F | 4.348056 | 0.3030414 | 0.003 |
| HFD vs HFD+H | 3.267347 | 0.2462698 | 0.003 |
| HFD vs H | 7.025273 | 0.4126379 | 0.003 |
| HFD+F vs HFD+H | 2.872975 | 0.2231788 | 0.003 |
| HFD+F vs S | 8.163847 | 0.4494558 | 0.003 |
| HFD+G vs HFD+SV | 7.205186 | 0.4446222 | 0.003 |
| HFD+G vs BS | 11.622515 | 0.5375191 | 0.003 |
| HFD+G vs H | 10.850136 | 0.5203868 | 0.003 |
| HFD+SG vs HFD+SU | 18.210354 | 0.6692436 | 0.003 |
| HFD+H vs SV | 14.015954 | 0.5836101 | 0.003 |
| HFD+S vs SV | 15.135553 | 0.6021571 | 0.003 |
| HFD+SU vs BS | 20.674672 | 0.7210081 | 0.003 |
| HFD+SV vs SU | 9.314711 | 0.5085918 | 0.003 |
| BS vs H | 2.664543 | 0.2103939 | 0.003 |
| BS vs SV | 18.723723 | 0.6518557 | 0.003 |
| H vs SU | 15.768763 | 0.6119333 | 0.003 |
| SU vs SV | 7.807105 | 0.4384264 | 0.003 |
| C vs HFD+SU | 8.783318 | 0.4939077 | 0.004 |
| C vs HFD+SV | 7.81386 | 0.4386394 | 0.004 |
| F vs H | 3.461123 | 0.25712 | 0.004 |
| SG vs HFD+F | 9.437082 | 0.4855195 | 0.004 |
| SG vs HFD+BS | 7.848429 | 0.4658256 | 0.004 |
| SG vs HFD+SV | 8.23399 | 0.4777762 | 0.004 |
| SG vs S | 8.258445 | 0.4523082 | 0.004 |
| HFD vs HFD+SV | 7.743533 | 0.462479 | 0.004 |
| HFD vs BS | 8.183063 | 0.4500376 | 0.004 |
| HFD+F vs HFD+SV | 9.245492 | 0.5067275 | 0.004 |
| HFD+F vs SV | 13.885558 | 0.581337 | 0.004 |
| HFD+G vs HFD+S | 4.753538 | 0.3221965 | 0.004 |
| HFD+BS vs HFD+H | 6.700568 | 0.4267723 | 0.004 |
| HFD+H vs HFD+S | 2.961128 | 0.2284622 | 0.004 |
| HFD+H vs S | 8.802322 | 0.4681508 | 0.004 |
| HFD+SU vs SV | 17.556432 | 0.6869673 | 0.004 |
| HFD+SV vs S | 14.485442 | 0.6167839 | 0.004 |
| BS vs SU | 15.661133 | 0.6103056 | 0.004 |
| S vs SV | 16.598624 | 0.6240407 | 0.004 |
| F vs HFD+SU | 7.630684 | 0.4881862 | 0.005 |
| F vs SV | 8.658893 | 0.4640625 | 0.005 |
| SG vs HFD+S | 10.205578 | 0.5050872 | 0.005 |
| HFD vs HFD+G | 4.631479 | 0.3165421 | 0.005 |
| HFD vs HFD+SU | 11.887317 | 0.5977336 | 0.005 |
| HFD vs S | 7.687578 | 0.4346315 | 0.005 |
| HFD+F vs HFD+S | 3.517418 | 0.2602137 | 0.005 |
| HFD+F vs SU | 12.506698 | 0.5556878 | 0.005 |
| HFD+G vs HFD+BS | 3.429167 | 0.2758967 | 0.005 |
| HFD+BS vs HFD+S | 2.894228 | 0.2433304 | 0.005 |
| HFD+BS vs SU | 11.122895 | 0.5527482 | 0.005 |
| HFD+BS vs SV | 13.153883 | 0.5937507 | 0.005 |
| HFD+S vs HFD+SU | 12.589455 | 0.6114516 | 0.005 |
| HFD+S vs S | 8.512955 | 0.4598377 | 0.005 |
| HFD+SU vs S | 17.711487 | 0.688855 | 0.005 |
| HFD+SU vs SU | 13.264738 | 0.6237903 | 0.005 |
| HFD+SV vs BS | 13.982769 | 0.6084023 | 0.005 |
| HFD+SV vs H | 15.32613 | 0.6300275 | 0.005 |
| G vs HFD+BS | 6.69954 | 0.4267348 | 0.006 |
| SG vs HFD+SU | 11.930525 | 0.5986057 | 0.006 |
| HFD vs HFD+BS | 2.720936 | 0.2321432 | 0.006 |
| HFD+G vs HFD+SU | 9.621169 | 0.5460006 | 0.006 |
| HFD+H vs HFD+SU | 11.97206 | 0.5994404 | 0.006 |
| HFD+H vs HFD+SV | 9.207683 | 0.5057032 | 0.006 |
| HFD+SU vs H | 20.69876 | 0.7212423 | 0.006 |
| SG vs HFD+H | 9.433884 | 0.4854348 | 0.007 |
| HFD+G vs S | 10.176667 | 0.504378 | 0.007 |
| HFD+BS vs HFD+SU | 10.579848 | 0.6018168 | 0.007 |
| HFD+F vs HFD+SU | 11.035055 | 0.5797228 | 0.008 |
| HFD+BS vs HFD+SV | 8.220729 | 0.5068039 | 0.011 |
| F vs HFD | 1.770213 | 0.1503977 | 0.012 |
| C vs HFD+BS | 2.573013 | 0.2046457 | 0.013 |
| HFD+SU vs HFD+SV | 9.355233 | 0.5720024 | 0.015 |
| F vs HFD+BS | 1.680381 | 0.1573335 | 0.025 |
| F vs HFD+S | 1.5014 | 0.1305406 | 0.081 |
| C vs F | 1.374099 | 0.1110464 | 0.136 |
