# Supplementary figures and images for: The development of metabolic endotoxemia is dependent on the type of sweetener and the presence of saturated fat in the diet
Source: Gut Microbes. 2020 Aug 17;12(1):1801301. doi: 10.1080/19490976.2020.1801301 (PMC7524302; doi:10.1080/19490976.2020.1801301)

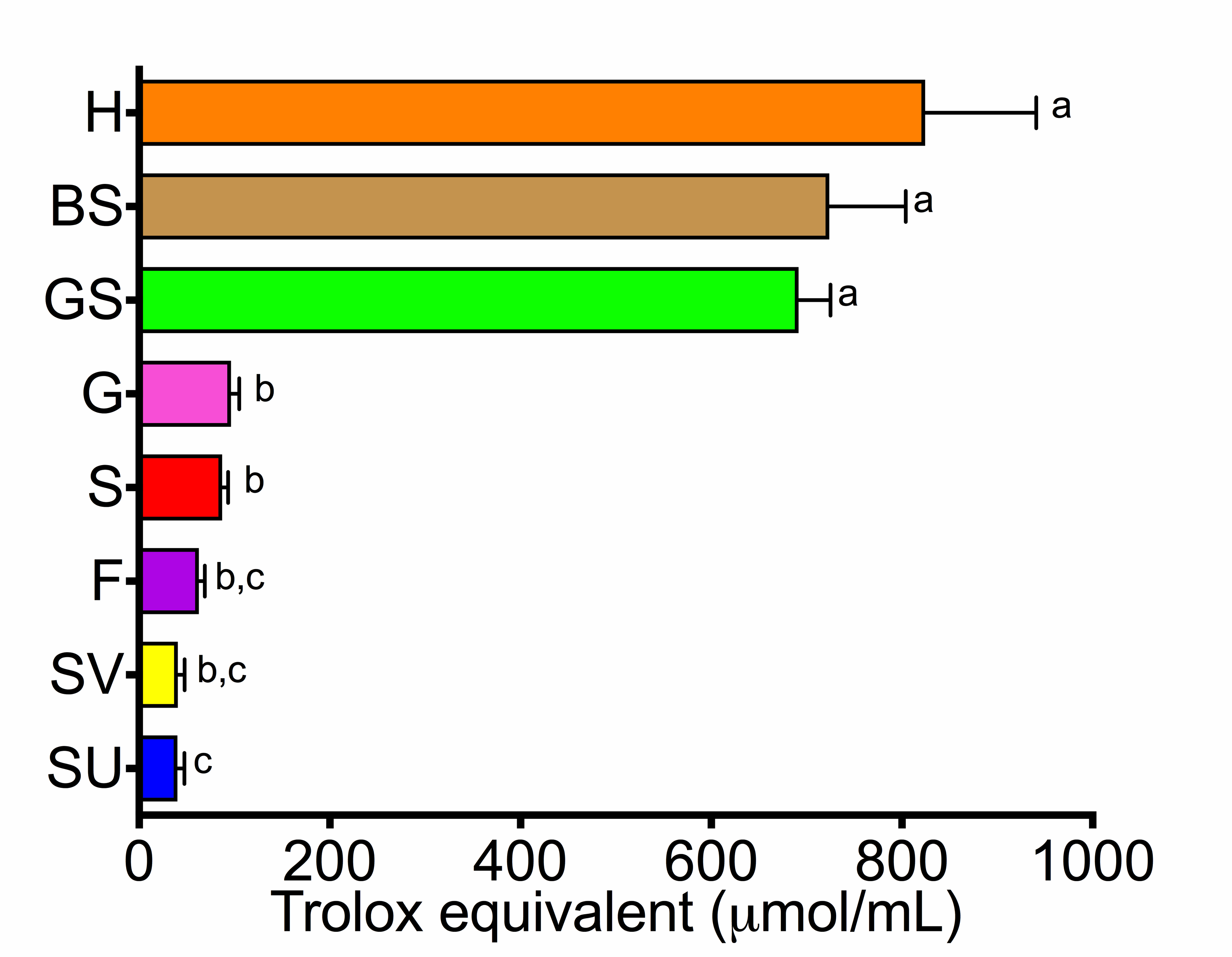

Supplement: Supplemental Material [file KGMI_A_1801301_SM9876.zip › Supplementary information/FIGURE S1.tiff]

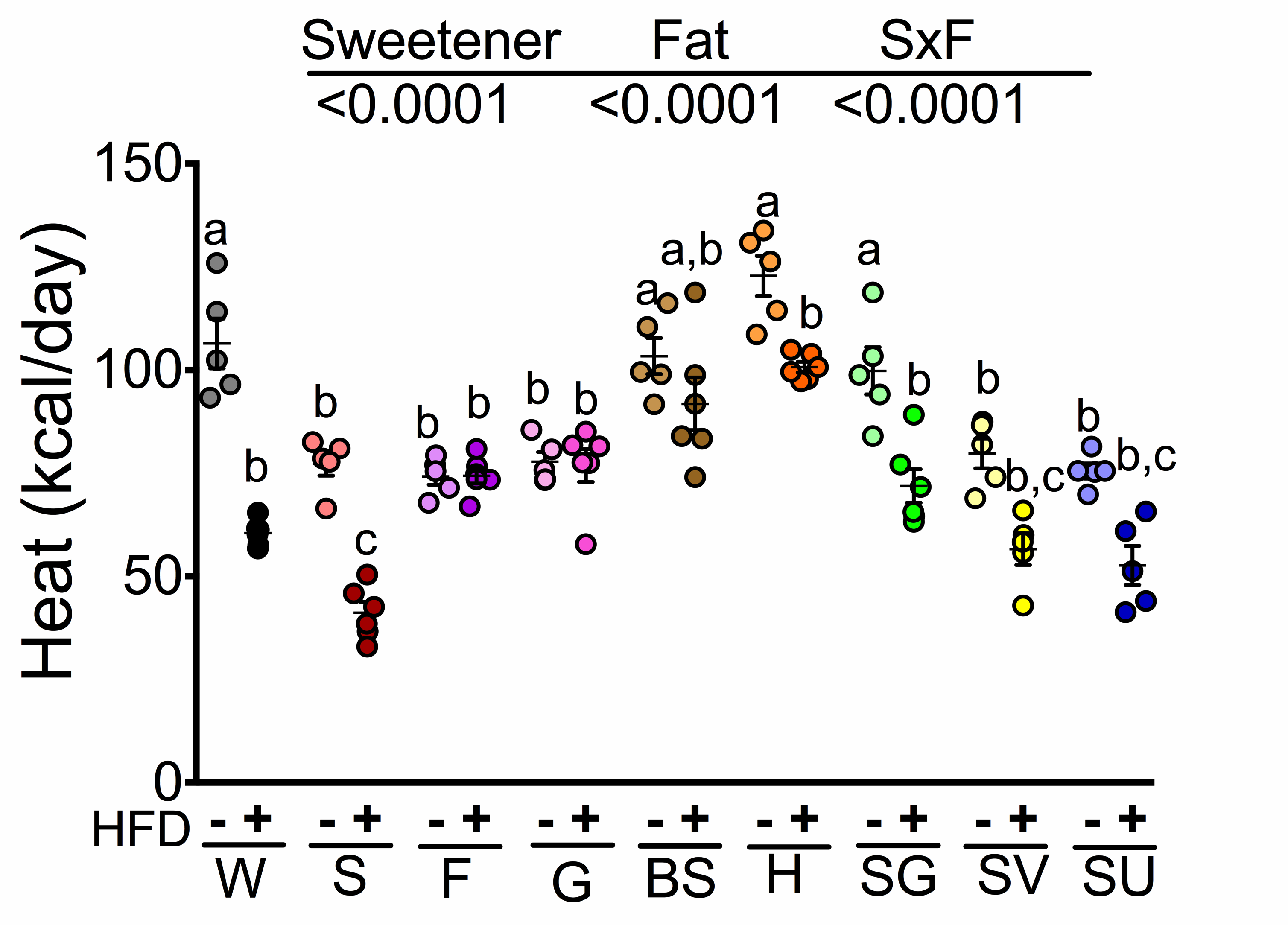

Supplement: Supplemental Material [file KGMI_A_1801301_SM9876.zip › Supplementary information/FIGURE S10.tiff]

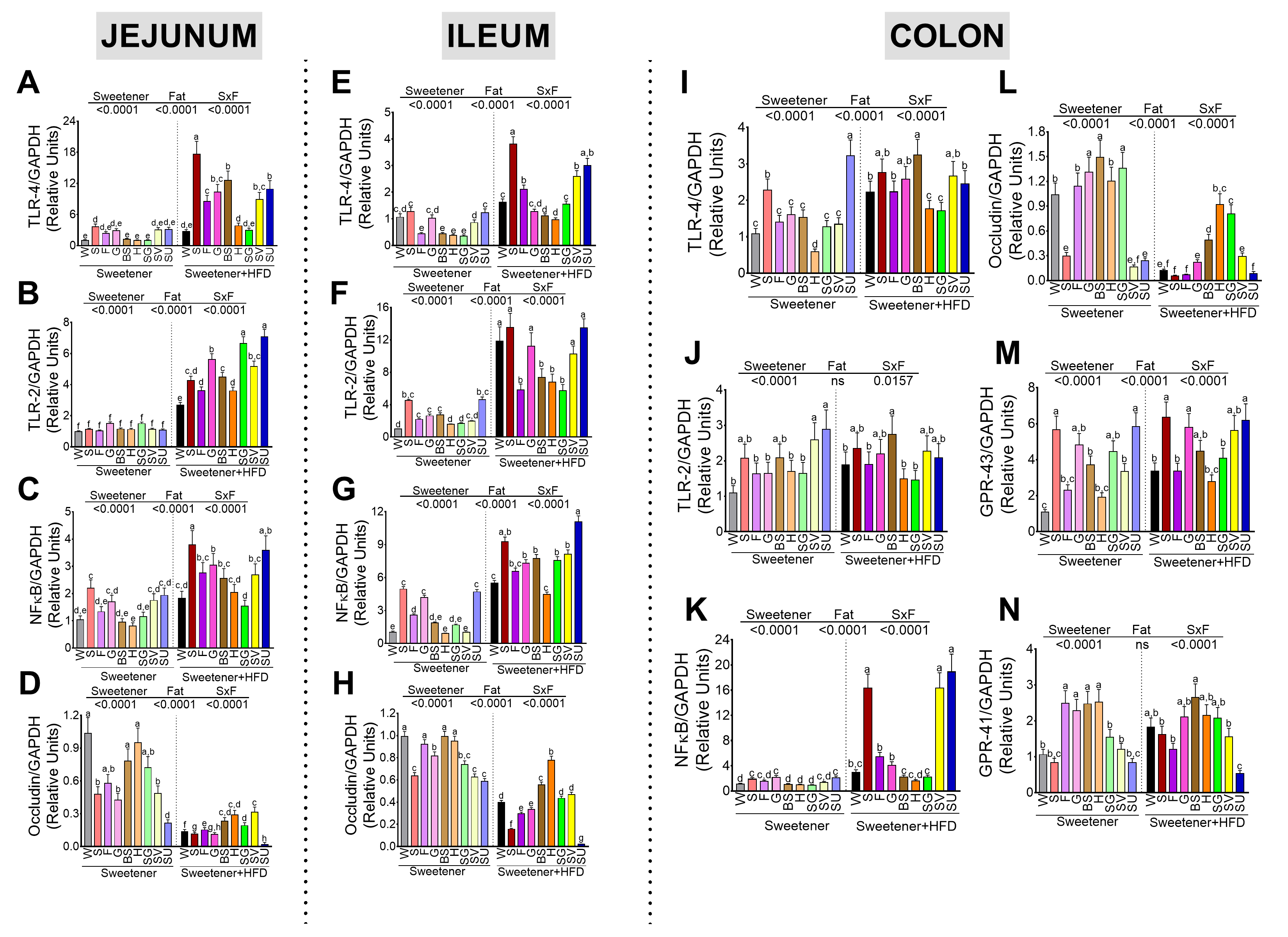

Supplement: Supplemental Material [file KGMI_A_1801301_SM9876.zip › Supplementary information/FIGURE S2.tif]

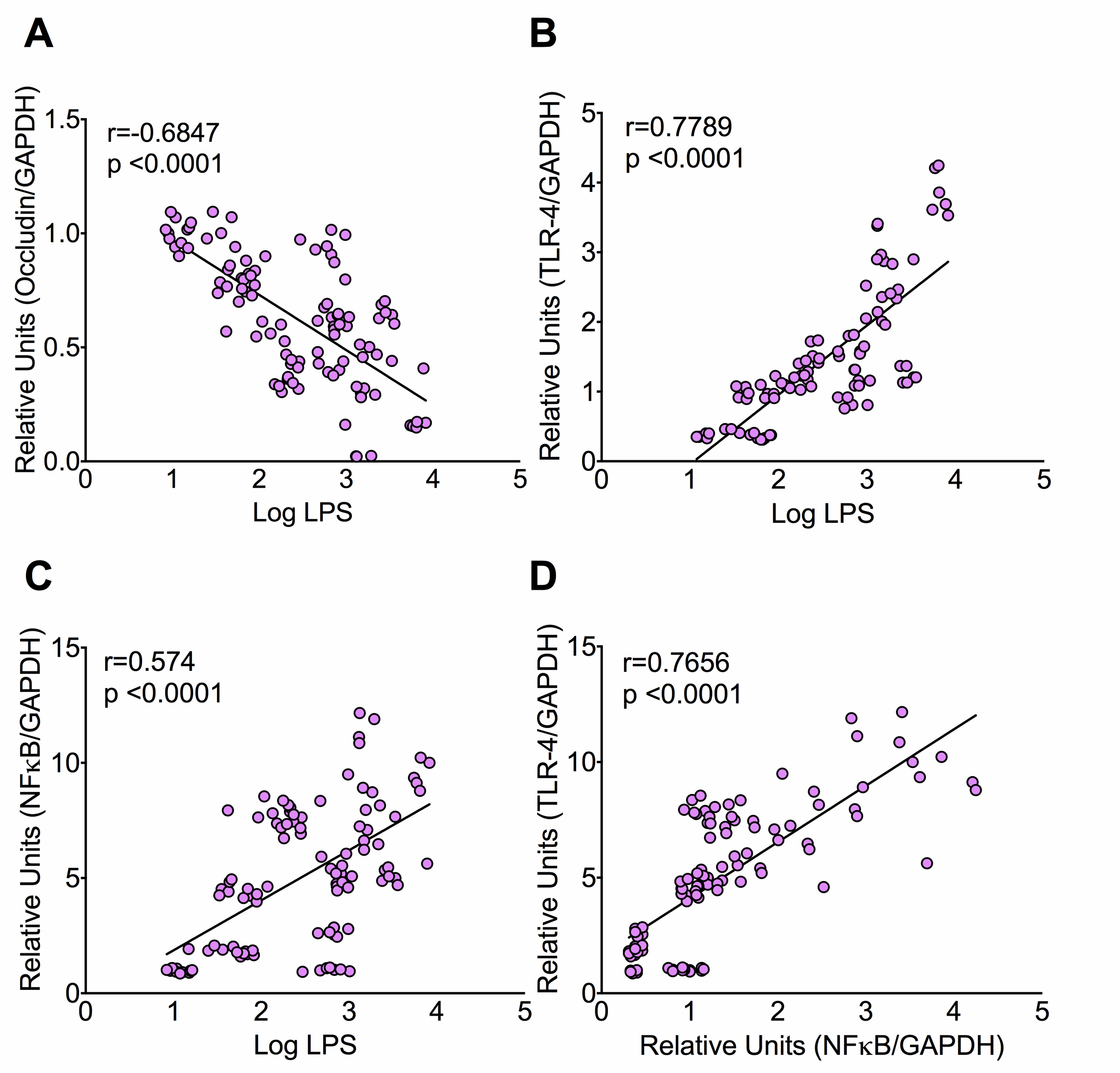

Supplement: Supplemental Material [file KGMI_A_1801301_SM9876.zip › Supplementary information/FIGURE S3.tiff]

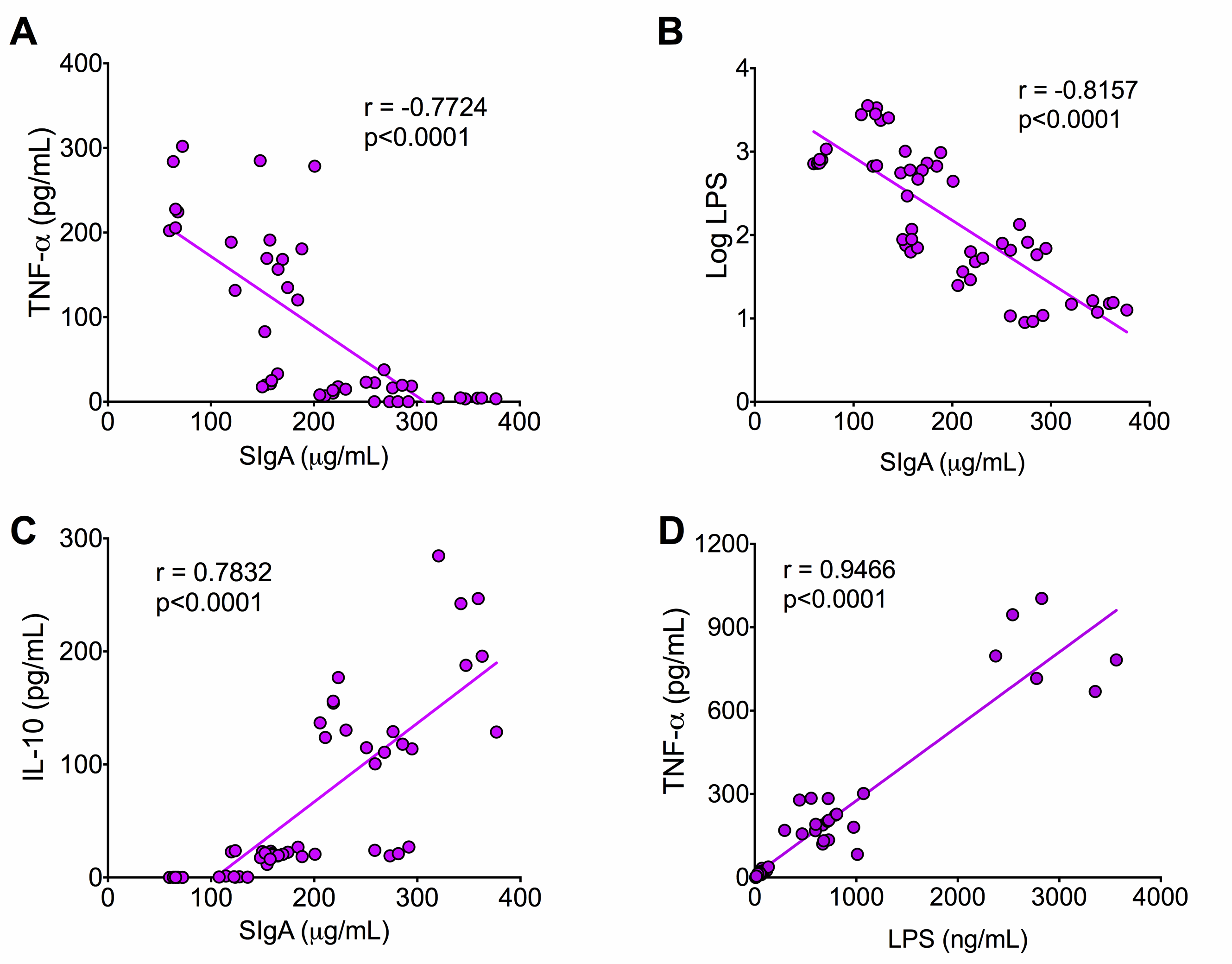

Supplement: Supplemental Material [file KGMI_A_1801301_SM9876.zip › Supplementary information/FIGURE S4.tiff]

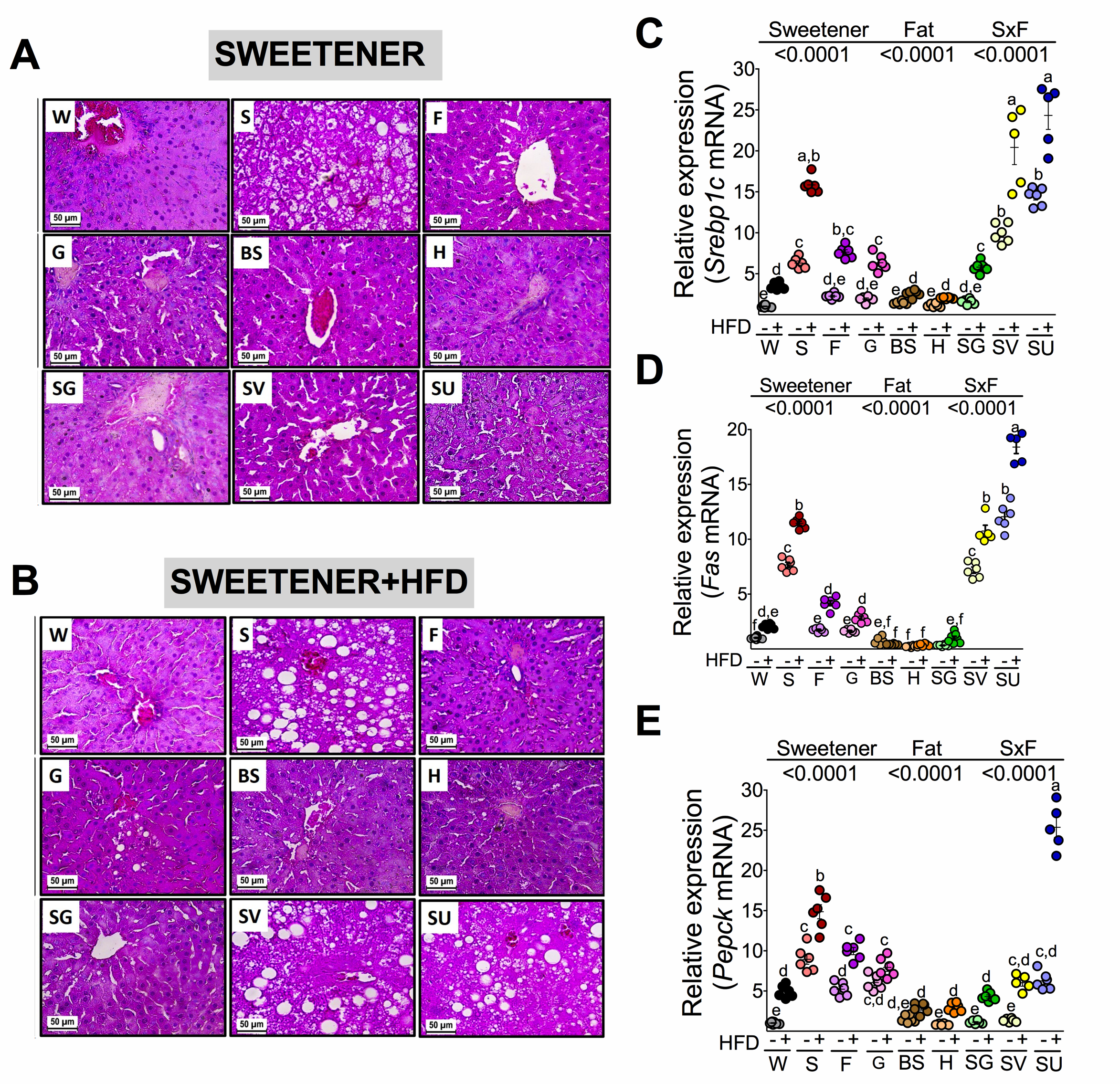

Supplement: Supplemental Material [file KGMI_A_1801301_SM9876.zip › Supplementary information/FIGURE S5.tiff]

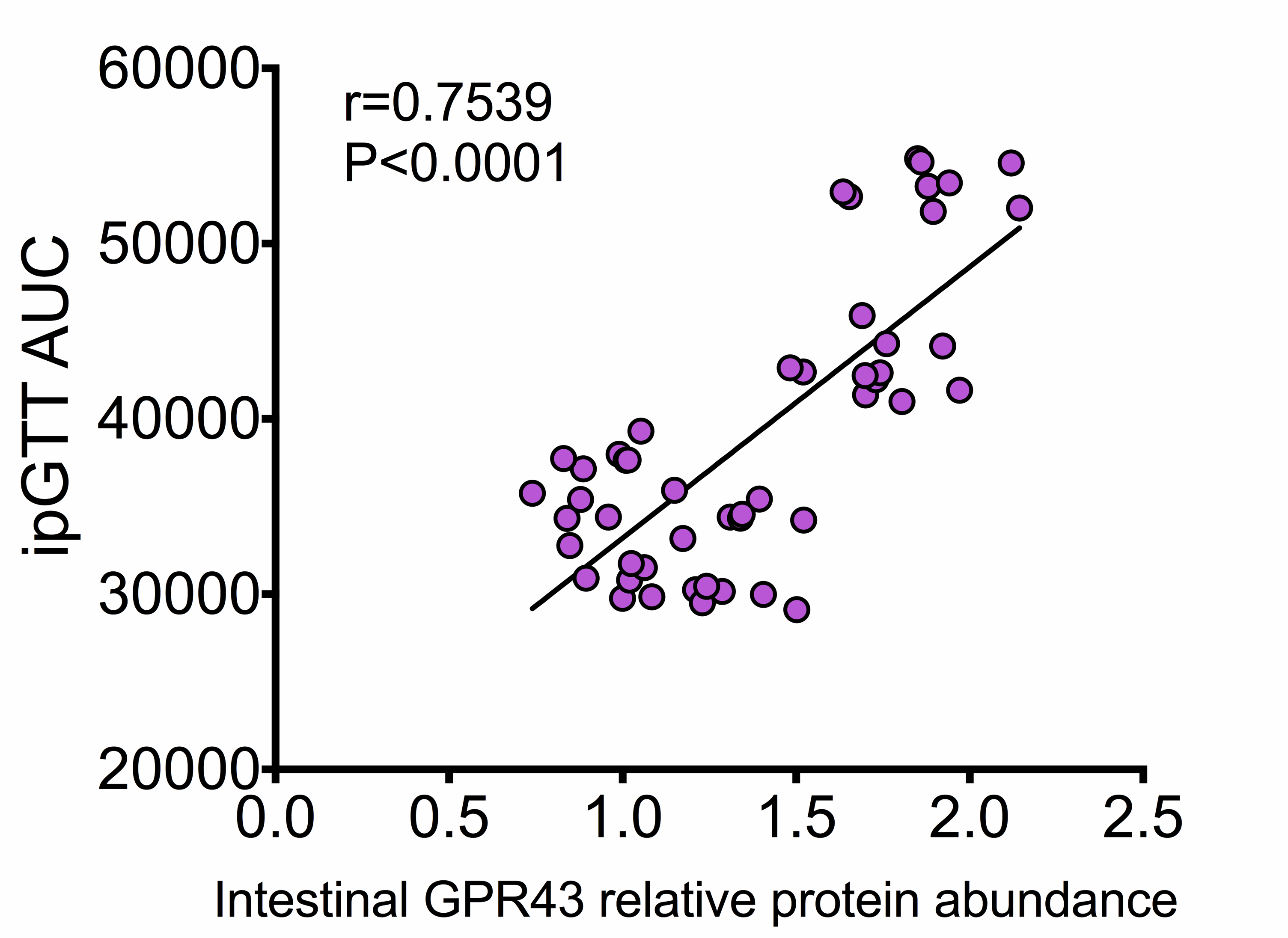

Supplement: Supplemental Material [file KGMI_A_1801301_SM9876.zip › Supplementary information/FIGURE S6.tiff]

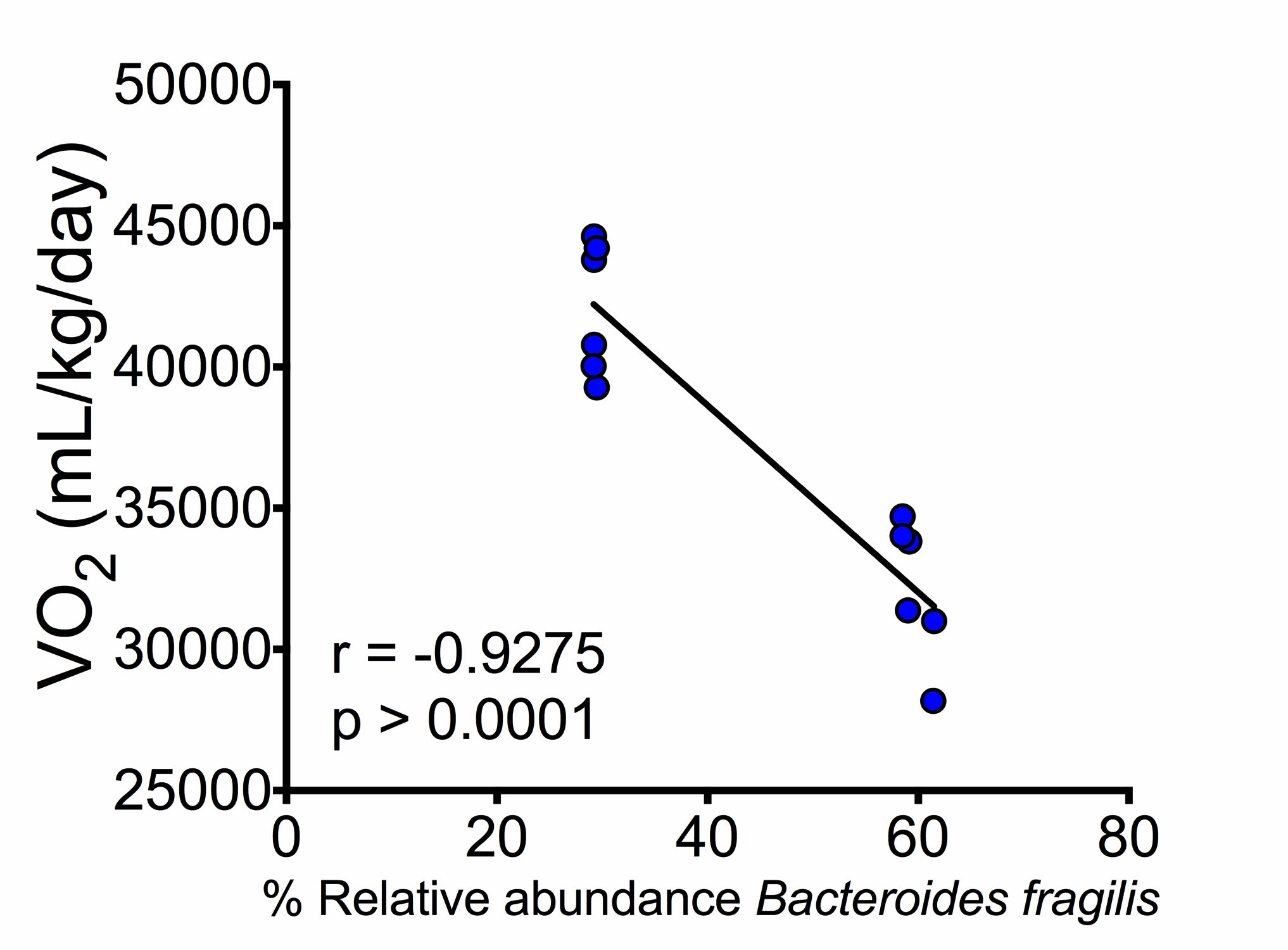

Supplement: Supplemental Material [file KGMI_A_1801301_SM9876.zip › Supplementary information/FIGURE S7.tiff]

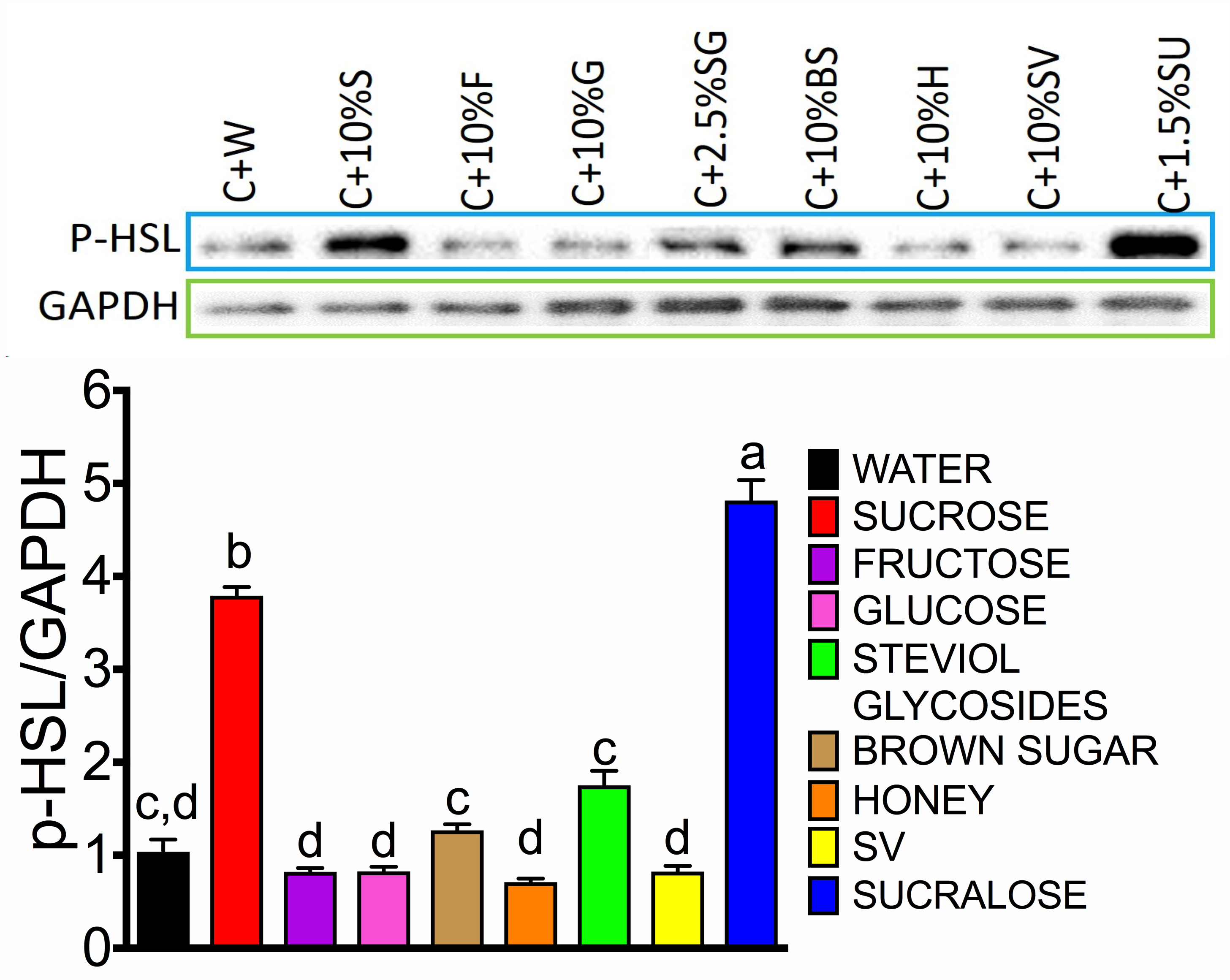

Supplement: Supplemental Material [file KGMI_A_1801301_SM9876.zip › Supplementary information/FIGURE S8.tiff]

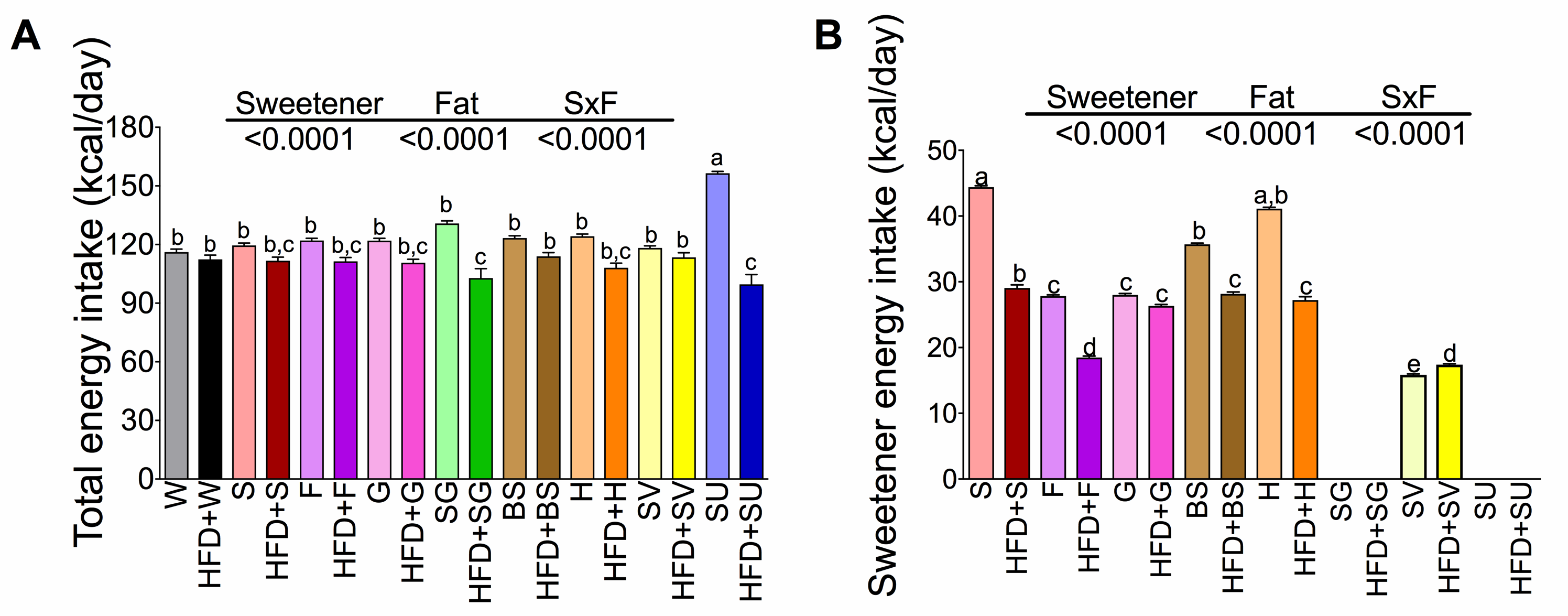

Supplement: Supplemental Material [file KGMI_A_1801301_SM9876.zip › Supplementary information/FIGURE S9.tiff]
